# Comparative effectiveness of GLP-1 receptor agonists versus mineralocorticoid receptor antagonists as fourth-line pharmacological therapy in patients with resistant hypertension and overweight or obesity: a retrospective multicenter cohort study in the USA

**DOI:** 10.1016/j.eclinm.2026.104176

**Published:** 2026-08-29

**Authors:** Zhejia Tian, Jonas M. Willerding, Kai M. Schmidt-Ott, Anette Melk, Bernhard M.W. Schmidt

**Affiliations:** a Department of Nephrology and Hypertension, Hannover Medical School, Hannover, Germany; b Department of Pediatric Kidney, Liver, Metabolic Diseases, and Neuropediatrics, Hannover Medical School, Hannover, Germany

**Keywords:** Resistant hypertension, GLP-1 receptor agonist, Mineralocorticoid receptor antagonist, Obesity, Cardiovascular outcome, Kidney health

## Abstract

**Background:**

Mineralocorticoid receptor antagonists (MRAs) are the guideline-recommended therapy for resistant hypertension. Resistant hypertension is particularly common in individuals with overweight or obesity. Glucagon-like peptide-1 receptor agonists (GLP-1RAs) induce substantial weight loss and reduce cardiovascular and renal events, with modest reductions in blood pressure. Whether GLP-1RAs provide benefit as an alternative therapeutic strategy in patients with resistant hypertension and overweight or obesity is unknown. We compared the effectiveness of GLP-1RAs and MRAs as fourth-line pharmacologic therapy in this population.

**Methods:**

In this retrospective multicenter cohort study using the TriNetX US Collaborative Network including 67 healthcare organizations, female and male adults with overweight or obesity and resistant hypertension (uncontrolled blood pressure despite ACE-inhibitors/angiotensin receptor blockers, calcium antagonists and diuretics) initiating a fourth-line pharmacological therapy between 01 June 2017 and 31 March 2025 were identified and included. Patients initiating GLP-1RAs (semaglutide or tirzepatide) were compared with those initiating MRAs (spironolactone or eplerenone). The primary outcome was major adverse cardiovascular events (MACE) during 2-year follow-up. Secondary outcomes included all-cause mortality, cardiovascular events, kidney outcomes, and blood pressure changes. Propensity score matching balanced baseline characteristics. Outcomes were analyzed using Kaplan–Meier estimates and Cox proportional hazards models.

**Findings:**

Among 213,309 eligible patients, 22,694 initiated GLP-1RAs and 5673 initiated MRAs. After propensity score matching, 4153 patients remained in each group. During a median follow-up of 1.4 years, GLP-1RA therapy was associated with lower risks of MACE (HR 0.63, 95% CI 0.52–0.78), all-cause mortality (HR 0.34, 95% CI 0.21–0.55), cardiovascular events (HR 0.74, 95% CI 0.59–0.92), major adverse kidney events (HR 0.64, 95% CI 0.46–0.88), and acute kidney injury (HR 0.62, 95% CI 0.46–0.83) compared with MRAs. Systolic blood pressure reductions at 12 weeks were similar (−5.7 [95% CI −4.0 to −7.4] mmHg versus −6.3 [95% CI −4.7 to −8.0] mmHg).

**Interpretation:**

In our retrospective study, among adults with resistant hypertension and overweight or obesity, GLP-1RA was associated with lower cardiovascular and kidney risk compared with MRAs despite smaller blood pressure reductions. GLP-1RAs may represent a potential alternative or complementary therapeutic option in this population. Prospective studies are needed to determine whether GLP-1RAs should be incorporated into treatment strategies for resistant hypertension in patients with overweight or obesity.

**Funding:**

None.

**Research in context**

**Evidence before this study**

We searched PubMed from January 1, 2000, through March 31, 2025, without language restrictions, using the term “resistant hypertension” AND (“mineralocorticoid receptor antagonist” OR “spironolactone” OR “eplerenone”) AND (“glucagon-like peptide-1 receptor agonist” OR “semaglutide” OR “tirzepatide”). Existing evidence indicates that resistant hypertension is a high-risk clinical condition frequently associated with overweight and obesity. Current international guidelines recommend mineralocorticoid receptor antagonists, like spironolactone, as the preferred fourth-line therapy. Recent large-scale trials have established that glucagon-like peptide-1 receptor agonists provide significant weight reduction, blood pressure lowering, and cardiovascular/kidney protection in patients with obesity or type II diabetes. To date, no studies have directly compared glucagon-like peptide-1 receptor agonists with mineralocorticoid receptor antagonists as fourth-line pharmacological therapy for resistant hypertension in adults with overweight or obesity.

**Added value of this study**

In this large-scale multicenter retrospective cohort study using real-world data from over 213,000 eligible patients, we compared for the first time the effectiveness of glucagon-like peptide-1 receptor agonists versus mineralocorticoid receptor antagonists in 4153 propensity-score-matched pairs with resistant hypertension and a BMI ≥27 kg/m^2^. Glucagon-like peptide-1 based therapy was associated with a significantly lower risk of major adverse cardiovascular events (HR 0.63, 95% CI 0.52–0.78) and a profound reduction in all-cause mortality (HR 0.34, 95% CI 0.21–0.55) compared with mineralocorticoid receptor antagonist therapy. Additionally, glucagon-like peptide-1 receptor agonists showed renal protection, by reducing the risk of major adverse kidney events and acute kidney injury. While both groups achieved significant blood pressure reduction at week 12, the cardiovascular and renal benefits of glucagon-like peptide-1 receptor agonists were more pronounced, likely mediated by pleiotropic metabolic effects.

**Implications of all the available evidence**

Our findings suggest that glucagon-like peptide-1 receptor agonists may serve as a highly effective alternative or complementary to mineralocorticoid receptor antagonists as fourth-line pharmacological therapy for the management of resistant hypertension in patients with overweight or obesity. The findings support a pleiotropic treatment strategy for hypertension in this population, targeting metabolic health in addition to blood pressure control. These data provide a strong rationale for future prospective studies to potentially update clinical guidelines for resistant hypertension.

## Introduction

Hypertension is a leading preventable cause of premature death and a major risk factor for adverse cardiovascular and renal outcomes. Resistant hypertension (RHTN) affects an estimated 10–20% of treated patients with hypertension and is associated with higher rates of end-organ damage, cardiovascular morbidity and mortality than controlled hypertension.^1^, ^2^, ^3^, ^4^ Patients with RHTN are frequently overweight or obese.^5^, ^6^, ^7^ Therefore, treatment strategies to target and maintain sustained weight loss should be incorporated into the treatment regimen, as weight regain is common even with intensive lifestyle modification.^8^

Glucagon-like peptide-1 receptor agonists (GLP-1RAs) have been extensively studied in recent years, demonstrating robust, favorable effects on weight reduction, blood pressure (BP) lowering, and cardiovascular and renal outcomes.^9^, ^10^, ^11^, ^12^, ^13^, ^14^, ^15^, ^16^, ^17^, ^18^, ^19^ These benefits suggest that GLP-1RAs hold promise as an appealing pharmacological option for the treatment of RHTN,^20^ especially in individuals with overweight or obesity.

For resistant hypertension, current evidence and guideline recommendations support adding mineralocorticoid receptor antagonists (MRAs), such as spironolactone or eplerenone, as fourth-line therapy after optimization of a three-drug regimen.^1^^,^^5^^,^^21^, ^22^, ^23^ In obesity, increased secretion of trophic factors from visceral adipose tissue may promote aldosterone excess and subsequent mineralocorticoid receptor overactivation.^24^ Accordingly, MRAs have long been hypothesized to confer greater BP-lowering effects in obesity-related hypertension.^25^

In patients with RHTN and overweight or obesity, an important clinical question is whether treatment should follow the established approach of initiating an MRA as fourth-line therapy, or whether GLP-1RAs may also be considered given their potential effects on weight reduction, BP reduction, and cardiovascular risk. To evaluate the potential cardiovascular and renal benefits of GLP-1RAs as a fourth-line pharmacological therapy of RHTN, we performed a retrospective cohort study to compare the effectiveness of GLP-1RAs versus MRAs, the conventional fourth-line treatment for RHTN,^1^^,^^5^^,^^22^^,^^23^ in patients with RHTN and overweight or obesity.

## Methods

This retrospective observational study followed the Strengthening the Reporting of Observational Studies in Epidemiology reporting guideline.^26^

### Ethics

Ethics approval and informed consent were waived by the institutional review board at Hannover Medical School because the study was retrospective and used de-identified patient data. TriNetX is compliant with the Health Insurance Portability and Accountability Act.

### Data source and study population

The TriNetX US Collaborative Network collects de-identified patient-level data from 67 healthcare organizations nationwide across the United States, including electronic medical records from approximately 100 million patients.^27^ The available data included information about demographics, diagnoses (based on the International Classification of Diseases, Tenth Revision, Clinical Modification, ICD-10-CM codes), procedures (coded in The International Classification of Diseases, Tenth Revision, Procedure Coding System, ICD-10-PCS or Current Procedural Terminology, CPT), medication (coded in Veterans Affairs National Formulary), and laboratory tests (coded in Logical Observation Identifiers Names and Codes, LOINC).

Patients were included if they met the following criteria between June 2017 and March 2025: patients were aged 18 years and older; patients were diagnosed with RHTN either with ICD-10-CM code I1A.0 or with BP above goal (≥130/80 mmHg) despite treatment with 3 antihypertensive medications including an angiotensin-converting enzyme (ACE) inhibitor/angiotensin receptor blocker (ARB), a calcium antagonist and a diuretic according to the US guideline^1^; patients were prescribed with GLP-1RAs or MRAs at least 1 day after first incidence of RHTN; patients had a BMI above 27 kg/m^2^ and an estimated glomerular filtration rate above 30 ml/min/1.73 m^2^. Patients were excluded if they were prescribed both GLP-1RAs and MRAs during follow-up, had used GLP-1RAs or MRAs before the diagnosis of RHTN, had initiated another additional antihypertensive agent before GLP-1RA or MRA initiation or at baseline, had controlled BP (<130/80 mmHg) or systolic BP >180 mmHg at baseline, were pregnant during follow-up, had heart failure or another condition that could independently influence selection of the study medications, had secondary causes of resistant hypertension, had contraindications to GLP-1RAs or MRAs, or had died prior to study initiation (Supplemental Tables S1 and S2).

### Exposure and outcomes

Exposures were defined as initiation of GLP-1RAs (semaglutide or tirzepatide) in the intervention group or MRAs (spironolactone or eplerenone) in the comparator group. Consistent with a new-user design, both treatments were initiated as the first additional pharmacologic therapy after the standard three-drug antihypertensive regimen, consisting of an ACE inhibitor or ARB, a calcium antagonist, and a diuretic. Following an intention-to-treat-like framework, participants were classified according to the treatment initiated at baseline, irrespective of subsequent dose escalation, dose reduction, treatment discontinuation, or initiation of additional therapy.

The primary outcome was major adverse cardiovascular events (MACE), defined as a composite of acute or subsequent myocardial infarction, cardiac arrest, unstable angina, cerebral infarction, nontraumatic intracerebral hemorrhage, and all-cause mortality during 2-year follow-up time. Secondary outcomes comprised mortality, cardiovascular events included in MACE, acute kidney injury (AKI), and major adverse kidney events (MAKE), defined as dialysis dependence, end-stage kidney disease with eGFR <15 mL/min/1.73 m^2^ in accordance with the KDIGO guideline,^28^ or mortality. Adverse events were defined as gastrointestinal symptoms, infection, cholecystitis/acute pancreatitis, and orthostatic hypotension (Supplemental Tables S3 and S4). Moreover, Mean Changes in systolic BP, diastolic BP, and BMI were assessed at weeks 2, 4, 6, 8, 12, 16, 20, 24, 48, 72, and 96 after study initiation.

Mortality data were obtained from HCOs death records, billable codes from closed claims, Social Security Administration Master Death File, private obituaries, and private claims.

### Covariates

Baseline covariates were collected for inclusion in propensity score models to account for factors that could potentially confound the association between the exposure and the outcomes. These included sociodemographic characteristics, medical diagnoses, laboratory results, and medication use, as outlined in Table 1 and Supplemental Table S5. Sex was recorded as biological sex assigned at birth. TriNetX database obtained eGFR using the 2009 or 2021 creatinine-based CKD-EPI (CKD Epidemiology Collaboration) equation.^29^^,^^30^

**Table 1:** Baseline characteristics of study cohort before and after propensity score matching.

	Before PSM			After PSM		
	GLP-1RA users (N = 22,694)	MRA users (N = 5673)	Standardized difference	GLP-1RA users (N = 4153)	MRA users (N = 4153)	Standardized difference
Demographics						
Age, years	57.1 ± 10.8	59.7 ± 11.4	0.24	59.0 ± 10.8	58.9 ± 11.5	0.011
Female	11,505 (55.1%)	2174 (51.6%)	0.071	2268 (54.6%)	2230 (53.7%)	0.018
Male	9523 (44.9%)	2517 (48.4%)	0.071	1885 (45.4%)	1923 (46.3%)	0.018
White	12,107 (57.4%)	2089 (48.7%)	0.177	2139 (51.5%)	2129 (51.3%)	0.005
Black	7153 (34.4%)	1716 (40.7%)	0.129	1580 (38.0%)	1606 (38.7%)	0.013
Asian	448 (2.1%)	117 (2.8%)	0.046	117 (2.8%)	117 (2.8%)	<0.001
Medical records/comorbidities						
DM	14,028 (66.1%)	1784 (34.3%)	0.67	1717 (41.3%)	1704 (41.0%)	0.006
T1DM	556 (2.6%)	119 (2.3%)	0.021	111 (2.7%)	105 (2.5%)	0.009
T2DM	13,967 (65.8%)	1758 (33.8%)	0.675	1702 (41.0%)	1688 (40.6%)	0.007
Overweight and obesity	14,787 (69.7%)	2573 (49.5%)	0.42	2396 (57.7%)	2348 (56.5%)	0.024
CKD	2745 (12.9%)	803 (15.4%)	0.072	611 (14.7%)	605 (14.6%)	0.004
CIHD	2494 (11.7%)	750 (14.4%)	0.08	578 (13.9%)	567 (13.7%)	0.008
Atrial fibrillation and flutter	921 (4.3%)	328 (6.3%)	0.1	227 (5.5%)	219 (5.3%)	0.009
Peripheral artery disease	219 (1.0%)	65 (1.3%)	0.021	45 (1.1%)	48 (1.2%)	0.007
Cerebrovascular diseases	1503 (7.1%)	636 (12.2%)	0.175	438 (10.5%)	412 (9.9%)	0.021
Gout	1285 (6.1%)	405 (7.8%)	0.069	297 (7.2%)	296 (7.1%)	0.001
Rheumatoid arthritis	430 (2.0%)	141 (2.7%)	0.045	108 (2.6%)	115 (2.8%)	0.01
Dyslipidemia	16,032 (75.5%)	3658 (70.4%)	0.116	2976 (71.7%)	2941 (70.8%)	0.019
COPD	1010 (4.8%)	311 (6.0%)	0.054	233 (5.6%)	228 (5.5%)	0.005
Hypertensive diseases	22119 (99.5%)	5183 (99.7%)	0.036	4139 (99.7%)	4138 (99.6%)	0.004
Hypertensive crisis	675 (3.2%)	397 (7.6%)	0.198	246 (5.9%)	229 (5.5%)	0.018
CTD	411 (1.9%)	140 (2.7%)	0.05	111 (2.7%)	110 (2.6%)	0.001
Neoplasms	6765 (31.9%)	1838 (35.4%)	0.074	1444 (34.8%)	1434 (34.5%)	0.005
Hepatic fibrosis	129 (0.6%)	48 (0.9%)	0.036	41 (1.0%)	33 (0.8%)	0.021
Nicotine dependence	2916 (13.7%)	853 (16.4%)	0.075	604 (14.5%)	625 (15.0%)	0.014
Alcohol related disorders	760 (3.6%)	386 (7.4%)	0.169	239 (5.8%)	242 (5.8%)	0.003
Medication						
Insulin	6354 (29.9%)	931 (17.9%)	0.285	878 (21.1%)	859 (20.4%)	0.017
Dapagliflozin	1204 (5.7%)	83 (1.6%)	0.219	72 (1.7%)	78 (1.9%)	0.011
Empagliflozin	3249 (15.3%)	244 (4.7%)	0.359	252 (6.1%)	236 (5.7%)	0.016
Canagliflozin	381 (1.8%)	26 (0.5%)	0.116	30 (0.7%)	26 (0.6%)	0.012
HMG CoA reductase inhibitors	14,220 (67.0%)	3170 (61.0%)	0.125	2551 (61.4%)	2520 (60.7%)	0.015
Atorvastatin	9931 (46.8%)	2293 (44.1%)	0.054	1855 (44.7%)	1794 (43.2%)	0.03
Aspirin	5516 (26.0%)	1685 (32.4%)	0.142	1294 (31.2%)	1248 (30.1%)	0.024
Laboratory measurement						
BMI, kg/m^2^	38.5 ± 7.9	34.3 ± 7.0	0.56	36.3 ± 7.7	35.1 ± 7.0	0.151
HbA1c, %	7.1 ± 1.8	6.2 ± 1.2	0.57	6.7 ± 1.7	6.2 ± 1.3	0.292
SBP, mmHg	136.9 ± 17.7	145.3 ± 19.6	0.45	140.3 ± 18.1	144.2 ± 19	0.215
DBP, mmHg	81.5 ± 12.3	82.2 ± 12.9	0.06	81.7 ± 12.6	82.1 ± 12.8	0.029
eGFR, ml/min/1.73 m^2^	83.9 ± 21.4	79.9 ± 20.8	0.19	80.9 ± 21.2	80.7 ± 20.8	0.007
UACR, mg/g creatinine	70.3 ± 166	89.2 ± 193	0.1	94.9 ± 189	87.7 ± 189	0.04
CRP, mg/l	22.3 ± 44.6	23.1 ± 45.0	0.02	21.4 ± 46.0	23.4 ± 46.2	0.044
Hemoglobin, g/dl	13.8 ± 1.7	13.5 ± 1.8	0.15	13.7 ± 1.7	13.5 ± 1.8	0.104
LDL Cholesterol, mg/dl	96.1 ± 37.0	100.0 ± 38.6	0.1	100.1 ± 37.6	99.2 ± 38.7	0.024
HDL Cholesterol, mg/dl	46.5 ± 13.5	49.4 ± 15.1	0.21	49.0 ± 14.8	48.9 ± 14.8	0.011

Means are presented as estimates with standard deviation. Proportions are presented as estimates with 95% confidence intervals.

BMI, body mass index; CIHD, chronic ischemic heart disease; CKD, chronic kidney disease; COPD, chronic obstructive pulmonary disease; CRP, c-reactive protein; CTD, connective tissue disease; DBP, diastolic blood pressure; DM, diabetes mellitus; eGFR, estimated glomerular filtration rate; GLP-1RA, glucagon-like peptide-1 receptor agonist; HbA1c, hemoglobin A1C; HDL, high-density lipoprotein; HR, hazard ratio; LDL, low-density lipoprotein; MRA, mineralocorticoid receptor antagonist; PSM, propensity score matching; SBP, systolic blood pressure; T1DM, type 1 diabetes mellitus; T2DM, type 2 diabetes mellitus; UACR, urine albumin creatinine ratio.

### Statistical analysis

Baseline characteristics were summarized as means with standard deviation and as counts with frequency or proportion. Two-sample t-test was applied to compare the means of change in BP and BMI. The use of parametric tests is justified by the patient numbers considering central-limit theorem.^31^ Propensity scores were estimated using logistic regression incorporating all baseline covariates. One-to-one propensity score matching (PSM) was then performed using a greedy nearest-neighbor algorithm with a caliper of 0.1 pooled standard deviations to achieve balance between the cohorts. Variables were considered adequately balanced if the standardized mean difference was less than 0.1. After matching, survival probabilities were estimated with the Kaplan–Meier method, while outcome differences were evaluated using the log-rank test and Cox proportional hazards models. *P* values and hazard ratios (HR) with their corresponding 95% confidence intervals (CI) were reported. To assess the robustness of the observed association to potential unmeasured confounding, we calculated post-hoc E-values for the primary and secondary outcomes. The E-value estimates the minimum strength of association that an unmeasured confounder would need to have with both treatment assignment and the outcome, conditional on the measured covariates, to fully explain away the observed association.^32^ For Cox regression, missing data of covariates were imputed by the TriNetX build-in statistical tool. Participants were censored the day after their last clinical record within the follow-up period.

Prespecified subgroup analyses were conducted, stratified by sex (female and male), race (White, Black or African American, and Asian), baseline age (18–49, 50–64, and ≥65 years), baseline BMI (27–29.9, 30–34.9, ≥35 kg/m^2^), baseline systolic BP (130–139, 140–159, ≥160 mmHg), baseline eGFR (≥60, 45–59.9, and 30–44.5 ml/min/1.73 m^2^), baseline hemoglobin (<10 g/dl and ≥10 g/dl), and comorbidities (diabetes mellitus, chronic kidney disease, and dyslipidemia).

In our study, tirzepatide and semaglutide were combined into a single intervention group. To assess potential heterogeneity between the two agents, we performed an additional prespecified analysis comparing their associations with primary and secondary outcomes. In addition, to explore the potential complementary effect of GLP-1 RAs and MRAs, we performed a post-hoc analysis comparing concurrent initiation of MRAs plus GLP 1-RAs and MRAs monotherapy.

Several prespecified sensitivity analyses were conducted: first, the study duration was extended to five years to ensure adequate evaluation of long-term effects. Second, patients with heart failure were also included, given that the cardiovascular benefits of MRAs have been primarily established in patients with heart failure. As post-hoc sensitivity analyses, we first performed an analysis without excluding patients who subsequently initiated the comparator therapy, in which patients were classified according to their baseline treatment and retained in their original groups regardless of subsequent comparator initiation. Second, patients with missing values for any baseline covariates included in the propensity score model were excluded. Third, to evaluate whether residual imbalances after PSM affected our findings, we performed a multivariable Cox proportional hazards analysis adjusted for all covariates included in the propensity score model.

Robustness of the main findings was further evaluated using prespecified positive and negative control analyses, including a negative exposure control (vitamin C supplementation), a positive outcome control (hyperkalemia, a known adverse effect of MRAs), and negative outcome controls (traumatic subdural hematoma, skin cancer, headache, and conjunctivitis).

All tests were 2-sided, and *P* < 0.05 was considered statistically significant for hypothesis testing. Statistical analyses were conducted using either the built-in statistical tools of the TriNetX research platform (Java 11.0.16; R 4.0.2; Python 3.7) or R version 4.3.2.

### Role of the funding source

No funding was received for this work.

## Results

**Fig. 1:**
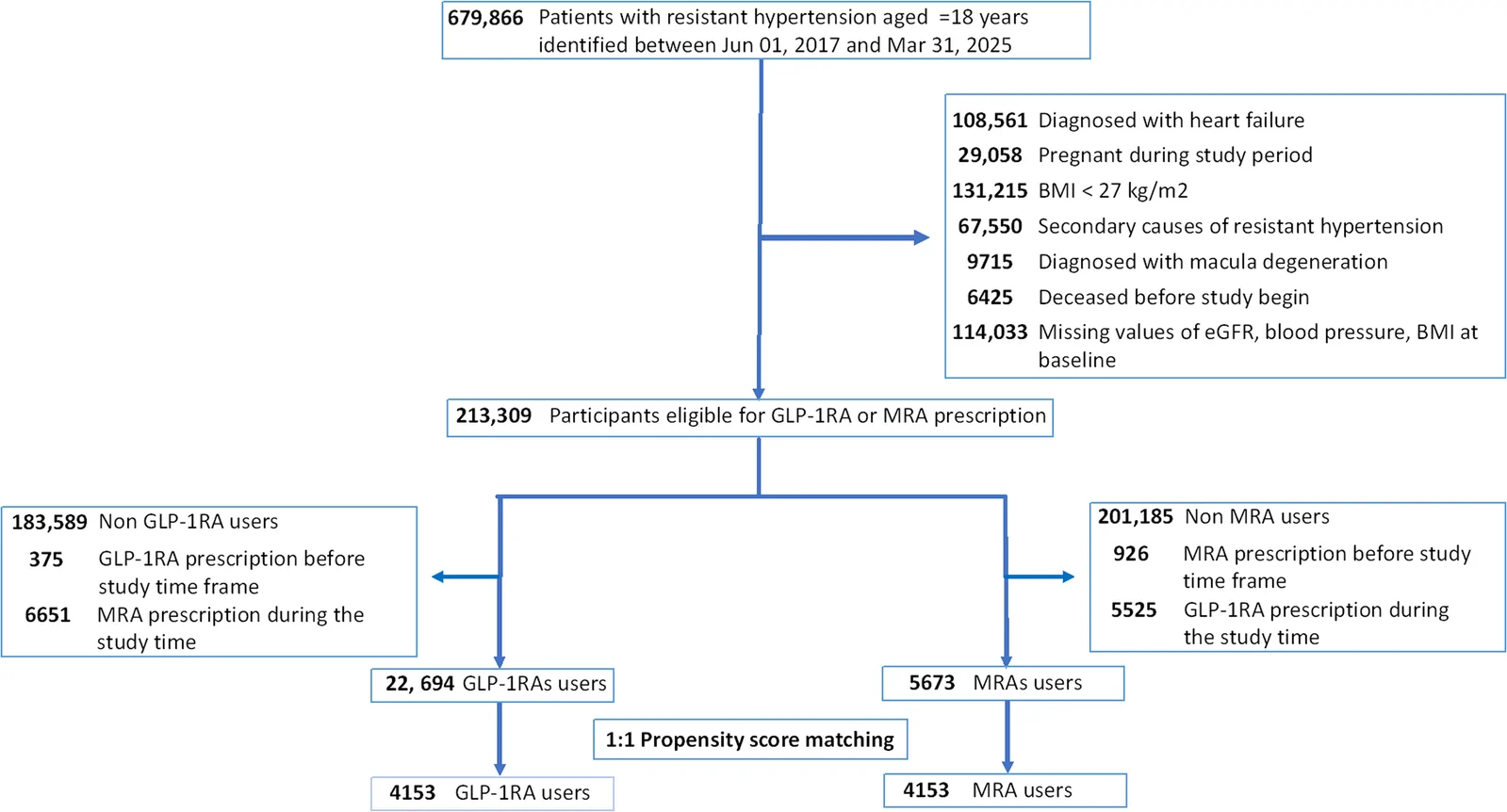
**Study profile.** Abbreviations: BMI, body mass index; eGFR, estimated glomerular filtration rate; GLP-1RA, glucagon-like peptide-1 receptor agonist; MRA, mineralocorticoid receptor antagonist.

Of 679,866 patients with resistant hypertension assessed for eligibility, 213,309 met the inclusion criteria and were included in the final analysis. 22,694 initiated GLP1-1RAs and 5673 initiated MRAs following the first diagnosis of RHTN (Fig. 1). Prior to matching, GLP-1RA-treated patients were younger with a mean (SD) age of 57.1 (10.8) years compared to MRA-treated patients with a mean age of 59.7 (11.4) years. Among GLP-1RA users, 11,505 (55.1%) of 22,694 were female, 12,107 (57.4%) of 22,694 were White, and 7153 (34.4%) of 22,694 were Black. In comparison, among MRA users, 2174 (51.6%) of 5673 were female, 2089 (48.7%) of 5673 were White, and 1716 (40.7%) of 5673 were Black. GLP-1RA users exhibited a higher prevalence of diabetes, obesity, chronic ischemic heart disease and had higher baseline BMI and eGFR. After matching, baseline sociodemographic characteristics, comorbidities, medication use, and most laboratory parameters were well balanced between the groups, with 4153 patients in each group (Table 1).

**Fig. 2:**
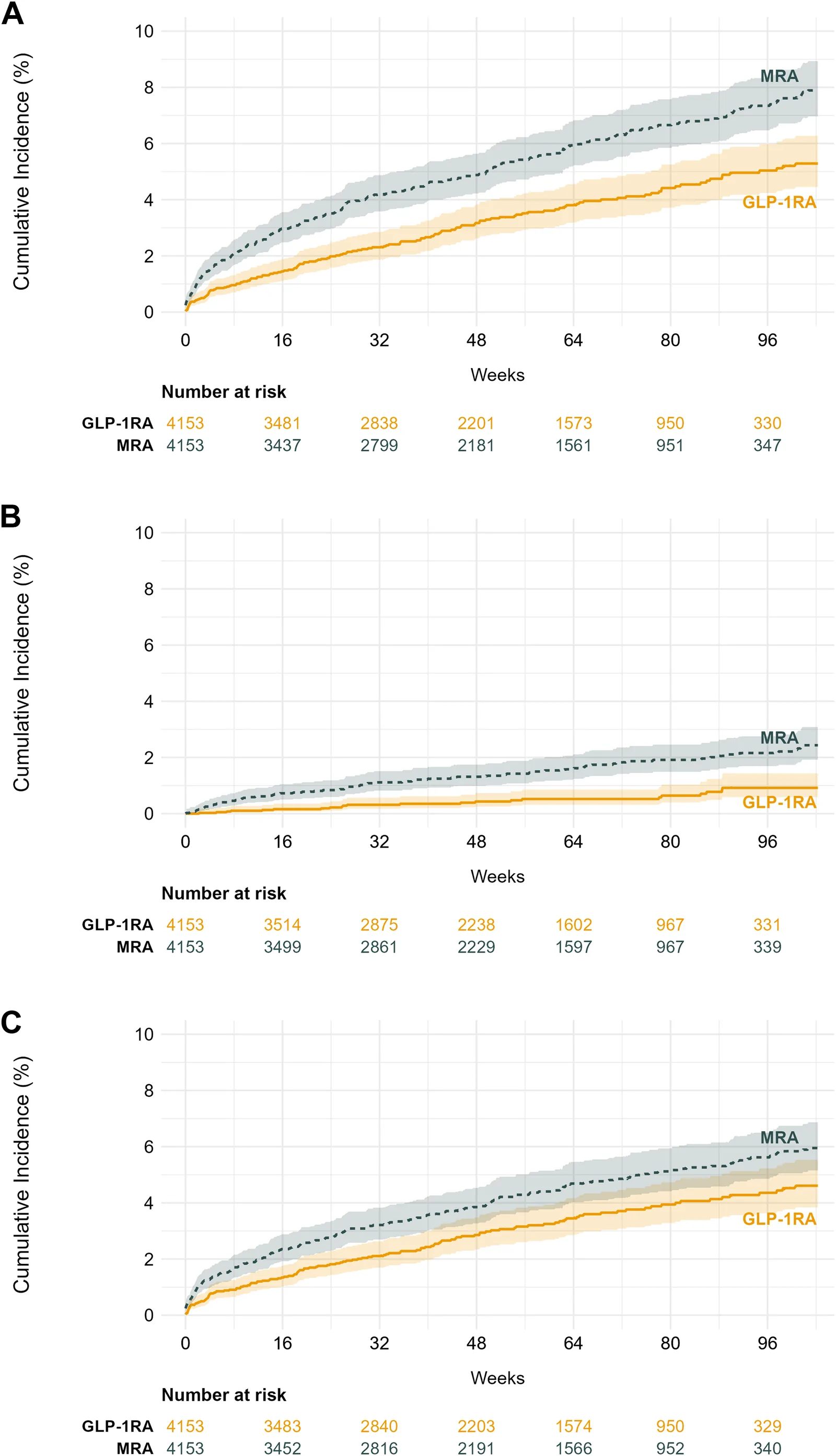
**Cumulative incidence of primary and secondary outcomes throughout the study period.** Panels A, B, and C depict cumulative incidence curves for MACE (A), all-cause mortality (B), and cardiovascular events (C), respectively, with 95% confidence intervals. Number at risk are shown below each plot. By the end of the study period, GLP-1RA therapy was associated with absolute risk reductions of −2.6% (95% CI −4.0% to −1.2%) for MACE, −1.5% (95% CI −2.3% to −0.7%) for all-cause mortality, and −1.3% (95% CI −2.6% to −0.1%) for cardiovascular events. Abbreviations: GLP-1RA, glucagon-like peptide-1 receptor agonist; MACE, major adverse cardiovascular event; MRA, mineralocorticoid receptor antagonist.

**Fig. 3:**
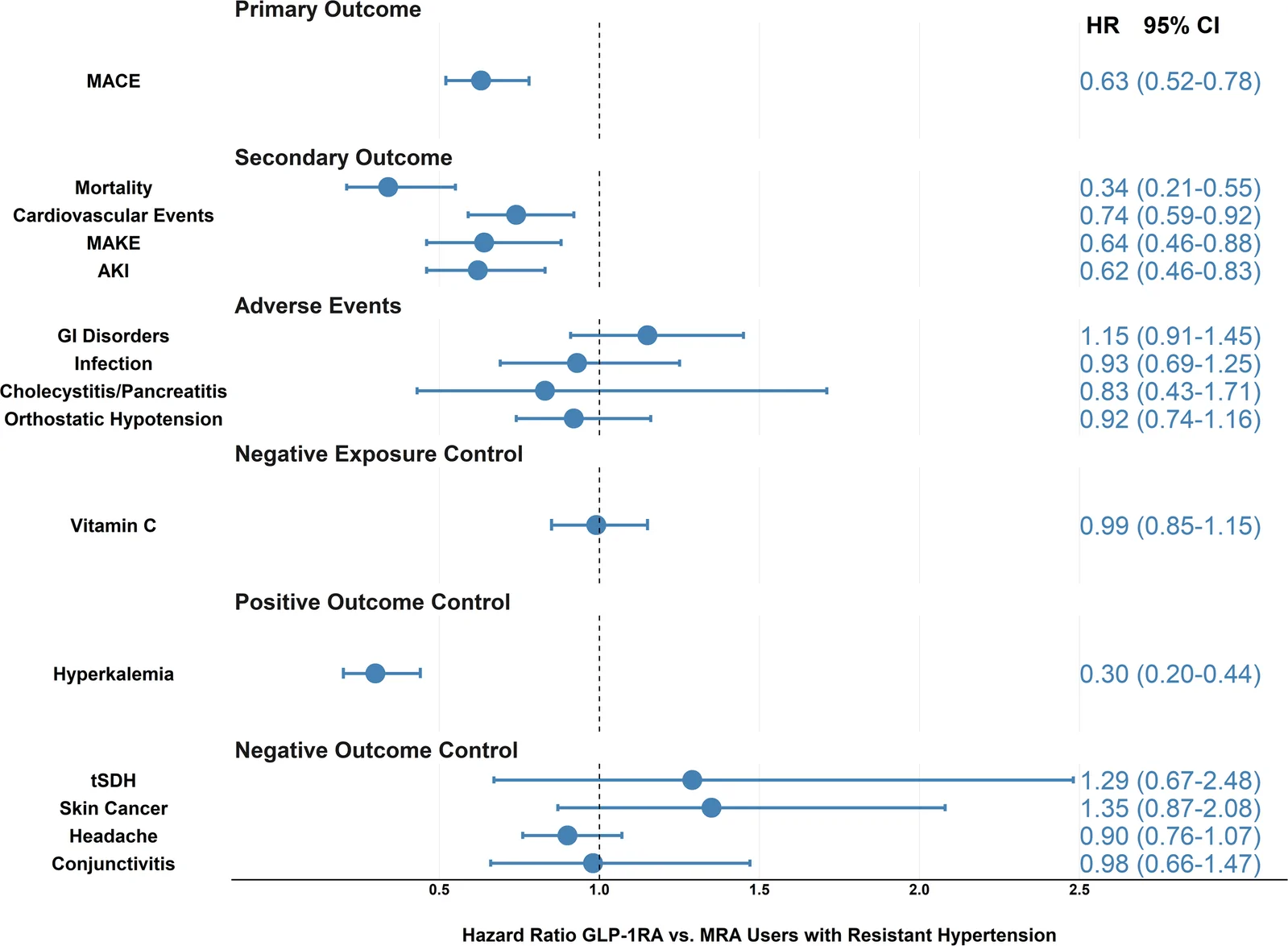
**Forest plot comparing GLP-1RAs and MRAs for clinical outcomes and control analyses in resistant hypertension with overweight or obesity.** Results are showed with hazard ratios (circles) and 95% confidence intervals (horizontal lines). Abbreviations: AKI, acute kidney injury; CI, confidence interval; GI, gastrointestinal; GLP-1RA, glucagon-like peptide-1 receptor agonist; HR, hazard ratio; MACE, major adverse cardiovascular event; MAKE, major adverse kidney event; MRA, mineralocorticoid receptor antagonist; tSDH, traumatic subdural hematoma.

During a median follow-up of 1.4 years, patients in the GLP-1RA group experienced a significantly lower incidence of MACE than those in the MRA group (3.5% [2.99 per 100 patient-years] versus 5.9% [4.48 per 100 patient-years]; log-rank *P* < 0.001) (Table 2). At the 2-year time point, GLP-1RA therapy resulted in an absolute risk reduction of −2.6% (95% CI −4.0% to −1.2%) (Fig. 2, Supplemental Table S6). Among patients in the GLP-1RA group, we observed an approximately 40% lower risk of MACE during the entire study period compared with the MRA group (HR 0.63, 95% CI 0.52–0.78) (Fig. 3). The risk of all-cause mortality was significantly lower in the GLP-1RA group (HR 0.34, 95% CI 0.21–0.55), as well as the risk of cardiovascular events (HR 0.74, 95% CI 0.59–0.92). Kidney endpoints showed a similar pattern, favoring the GLP-1RA group. MAKE occurred in 1.4% of patients in the GLP-1RA group and 2.4% of patients in the MRA group (HR 0.64, 95% CI 0.46–0.88; log-rank *P* = 0.007). In addition, AKI occurred in 1.7% of patients in the GLP-1RA group and 3.1% of patients in the MRA group (HR 0.62, 95% CI 0.46–0.83; log-rank *P* = 0.001) during the study period (Table 2 and Fig. 3). Across the primary and secondary outcomes, unmeasured confounding of substantial magnitude would be required to fully explain away the observed associations. The E-values for the point estimates and the confidence limits closest to the null were 2.55 [1.88] for MACE, 5.33 [3.04] for mortality, 2.04 [1.39] for cardiovascular events, 2.50 [1.53] for MAKE, and 2.61[1.70] for AKI, respectively.

**Table 2:** Primary and secondary outcomes.

Outcomes	GLP-1RAs		MRAs		HR (95% CI)	Log-rank *P*	E-value, point estimate [CI limit]
	Events	ER per 100 PY	Events	ER per 100 PY			
MACE	144	2.99 (2.52, 3.52)	247	4.48 (3.94, 5.07)	0.63 (0.52, 0.78)	<0.001	2.55 [1.88]
Mortality	22	0.46 (0.29, 0.69)	72	1.31 (1.02, 1.64)	0.34 (0.21, 0.55)	<0.001	5.33 [3.04]
Cardiovascular events	128	2.66 (2.22, 3.16)	188	3.41 (2.94, 3.93)	0.74 (0.59, 0.92)	0.007	2.04 [1.39]
MAKE	58	1.20 (0.91, 1.56)	101	1.83 (1.49, 2.22)	0.64 (0.46,0.88)	0.007	2.50 [1.53]
AKI	70	1.45 (1.13, 1.84)	125	2.27 (1.89, 2.70)	0.62 (0.46,0.83)	0.001	2.61 [1.70]

AKI, acute kidney injury; CI, confidence interval; ER, event rate; GLP-1RA, glucagon-like peptide-1 receptor agonist; HR, hazard ratio; MACE, major adverse cardiovascular events; MAKE, major adverse kidney events; MRA, mineralocorticoid receptor antagonist; PY, patient year.

Changes in systolic BP, diastolic BP, and BMI over the course of the study are illustrated in Supplemental Figure S1. BP lowering effects of GLP-1RAs or MRAs were evident as early as week two, with maximal effects observed at week 12. From study initiation to week 12, the mean change in systolic BP was −5.7 mmHg (95% CI −7.4 to −4.0) in the GLP-1RA group and −6.3 mmHg (95% CI −8.0 to −4.7]) (*P* = 0.63) in the MRA group. Corresponding mean changes in diastolic BP at week 12 were −2.2 mmHg (95% CI −3.4 to −1.0) mmHg for GLP-1RAs and −3.1 mmHg (95% CI −4.2 to −2.0) (*P* = 0.28) for MRAs. Body weight decreased continuously in the GLP-1RA group, with a mean change in BMI of −1.7 kg/m^2^ (95% CI −2.3 to −1.1) at week 96, corresponding to a 4.7% reduction in baseline body weight, whereas the magnitude of change was substantially smaller in the MRAs group (*P* < 0.001).

In prespecified subgroup analyses, GLP-1RAs were consistently associated with a lower risk of the primary outcome across female and male patients, other sociodemographic strata, age groups, BMI categories, systolic BP levels, kidney function, hemoglobin levels, and comorbidities when compared to MRAs (Table 3). Patients with male sex, higher baseline BMI, higher baseline systolic BP, or lower baseline eGFR were more likely to benefit from GLP-1RA therapy.

**Table 3:** Subgroup analysis of the primary outcome.

	GLP-1RAs		MRAs		HR	Log-rank *P*
	Events	ER per 100 PY	Events	ER per 100 PY		
Sex						
Women	107	3.42 (2.80, 4.13)	173	5.07 (4.34, 5.88)	0.67 (0.53,0.85)	<0.001
Men	97	3.90 (3.16, 4.75)	184	6.74 (5.80, 7.79)	0.57 (0.45,0.73)	<0.001
Race^a^						
White	157	4.31 (3.66, 5.04)	253	6.31 (5.56, 7.14)	0.67 (0.55,0.82)	<0.001
Black	90	3.79 (3.05, 4.66)	145	5.47 (4.61, 6.43)	0.66 (0.50,0.85)	0.002
Age, year						
18–49	42	3.98 (2.87, 5.39)	49	4.15 (3.07, 5.49)	0.89 (0.59,1.34)	0.566
50–64	143	4.47 (3.77, 5.27)	218	6.06 (5.28,6.92)	0.69 (0.56,0.86)	<0.001
≥65	84	4.29 (3.42, 5.32)	139	5.94 (5.00, 7.02)	0.66 (0.51,0.87)	0.003
BMI, kg/m^2^						
27–29.9	151	5.77 (4.89, 6.77)	220	7.13 (6.22, 8.14)	0.74 (0.60,0.91)	0.005
30–34.9	195	4.63 (4.01, 5.33)	316	6.45 (5.76, 7.21)	0.67 (0.56,0.80)	<0.001
≥35	202	4.23 (3.66, 4.86)	317	5.80 (5.18, 6.48)	0.68 (0.57,0.81)	<0.001
Systolic blood pressure, mmHg						
130–139	211	4.31 (3.75,4.94)	328	5.70 (5.10, 6.36)	0.70 (0.59,0.83)	<0.001
140–159	353	4.26 (3.82, 4.73)	536	5.59 (5.13,6.08)	0.71 (0.62,0.81)	<0.001
≥160	194	4.13 (3.57, 4.76)	320	5.82 (5.20, 6.50)	0.66 (0.55,0.78)	<0.001
eGFR, ml/min/1.73 m^2^						
≥ 60	263	4.46 (3.94, 5.04)	391	5.65 (5.11, 6.24)	0.73 (0.62,0.85)	<0.001
45–59.9	184	6.34 (5.45, 7.32)	266	7.89 (6.97, 8.90)	0.74 (0.62,0.90)	0.002
30–44.9	92	7.33 (5.91, 8.99)	147	10.35 (8.74, 12.17)	0.66 (0.51, 0.85)	0.002
Hemoglobin, g/dl						
<10	65	6.33 (4.89, 8.08)	94	8.25 (6.67, 10.09)	0.72 (0.52, 0.99)	0.04
≥10	141	3.77 (3.18, 4.45)	229	5.35 (4.68, 6.10)	0.67 (0.54, 0.83)	<0.001
Diabetes mellitus						
With	89	3.94 (3.17, 4.86)	147	5.92 (5.01, 6.97)	0.63 (0.48,0.82)	<0.001
Without	51	2.19 (1.64, 2.89)	109	3.75 (3.08, 4.53)	0.53 (0.38,0.74)	<0.001
Chronic kidney disease						
With	47	5.10 (3.74, 6.79)	81	7.47 (5.93, 9.29)	0.64 (0.44, 0.91)	0.01
Without	78	2.87 (2.27, 3.59)	126	3.92 (3.27, 4.67)	0.67 (0.51, 0.89)	0.005
Dyslipidemia						
With	68	3.06 (2.39, 3.90)	113	4.39 (3.62, 5.29)	0.65 (0.48, 0.87)	0.004
Without	20	1.40 (0.92, 2.31)	37	2.29 (1.61, 3.15)	0.61 (0.35, 1.05)	0.07

BMI, body mass index; eGFR, estimated glomerular filtration rate; ER, event rate; GLP-1RA, glucagon-like peptide-1 receptor agonist; HR, hazard ratio; MRA, mineralocorticoid receptor antagonist; PY, patient year.

a Due to the small proportion of Asian participants, analysis of the primary outcome in this subgroup was not feasible.

No substantial differences were observed between tirzepatide and semaglutide for primary and secondary outcomes, with tirzepatide tending toward more favorable cardiovascular outcomes and semaglutide toward more favorable kidney outcomes (Supplemental Figure S2). In the analysis of concurrent initiation of both agents, there was a tendency to complementary effects of the combination (Supplemental Figure S3). Sensitivity analyses, including an extended study duration, inclusion of patients with heart failure, retention of patients who subsequently initiated the comparator therapy, exclusion of patients with missing baseline covariate data, and multivariable Cox proportional hazards adjustment, showed results consistent with the primary analysis (Supplemental Figure S4 and Supplemental Tables S7–S9).

In the positive outcome control analysis, GLP-1RA use was associated with a significantly lower risk of hyperkalemia compared with MRA use (HR 0.30, 95% CI 0.20–0.44; log-rank *P* < 0.001). In negative control analyses, vitamin C was used as a negative exposure, and traumatic subdural hematoma, skin cancer, headache, and conjunctivitis were each evaluated as negative outcome controls. No meaningful associations were observed in these analyses (Fig. 3).

Recorded adverse events were comparable between the two groups and are summarized in Fig. 3. Gastrointestinal disorders including vomiting, diarrhea, obstipation, and epigastric pain were numerically more frequently recorded in the GLP-1RA group, although the difference was not statistically significant (HR 1.15, 95% CI 0.91–1.45; log-rank *P* = 0.24).

## Discussion

In this retrospective multicenter cohort study utilizing clinical data, initiation of GLP-1RAs (semaglutide or tirzepatide) as fourth-line pharmacological therapy was associated with a significantly lower risk of MACE compared with initiation of MRAs (spironolactone or eplerenone) among patients with RHTN and overweight or obesity. This association remained consistent across extensive subgroup analyses accounting for sociodemographic characteristics, age, comorbidities, and laboratory measurements. Moreover, GLP-1RAs were also associated with a significant reduction in systolic BP, although the magnitude of effect was smaller than that observed with MRAs.

Patients with RHTN exhibit a substantially higher risk of cardiovascular events and kidney failure compared with those with controlled hypertension,^33^, ^34^, ^35^, ^36^ highlighting the critical need for optimized treatment strategies. Obesity is a well-established risk factor for RHTN, with more than half of the affected patients reported to be obese.^5^^,^^6^^,^^37^^,^^38^ Over the past decade, accumulating evidence from large-scale randomized controlled trials has shown that GLP-1RAs lower BP and confer cardiovascular and kidney benefits in individuals with overweight or obesity, irrespective of diabetes status.^9^, ^10^, ^11^, ^12^, ^13^, ^14^, ^15^ MRAs, recommended by current guidelines as the fourth-line agent for RHTN,^1^^,^^22^^,^^23^ improved cardiovascular and kidney health particularly among patients with heart failure.^39^, ^40^, ^41^ However, to date, no studies have directly evaluated the effects of GLP-1RAs and MRAs on the RHTN management. Our study provides novel and clinically important evidence addressing this gap.

In our study, GLP-1RAs were associated with a lower risk of cardiovascular and renal outcomes compared with MRAs across subgroups defined by sex, age, diabetes, chronic kidney disease as well as dyslipidemia status, baseline systolic BP, and baseline BMI in individuals with overweight or obesity and newly diagnosed with RHTN, despite greater BP reduction being observed in the MRA group. These effects may partially be mediated by sustained weight reduction in the GLP-1RA group. However, lifestyle intervention aimed at weight reduction has not yielded similarly significant reductions in cardiovascular events.^42^ Additionally, while GLP-1RAs optimize cardiometabolic risk factors, this optimization may only partially account for their observed kidney-protective effects.^43^ Within the complex cardiovascular-kidney-metabolic framework, GLP-1RAs may exert pleiotropic beneficial effects that extend beyond body weight reduction and diabetes management and may offer broader cardiovascular and renal benefits among patients with RHTN and overweight or obesity. Notably, GLP-1RAs and MRAs should not necessarily be considered mutually exclusive. In an exploratory analysis, concurrent initiation was associated with lower risks of mortality and kidney outcomes compared with MRA use alone, while the association with MACE was directionally favorable.

Our study extends prior evidence^17^^,^^19^^,^^44^^,^^45^ by demonstrating that GLP-1RAs significantly reduce systolic and diastolic BP by 5.7 mmHg and 2.2 mmHg, respectively, in patients with RHTN and overweight or obesity when used as a fourth-line pharmacological therapy. Meta-analyses assessing the antihypertensive effects of GLP-1RA-based therapies have reported similar results, with average reductions of 3–5 mmHg in systolic and 1–2 mmHg in diastolic BP on average.^17^^,^^18^^,^^44^^,^^46^ Mediation analyses indicated that more than 70% of the BP reduction effect of semaglutide and tirzepatide was attributable to weight loss.^17^^,^^19^ Beyond weight loss, several additional mechanisms have been studied to explain the antihypertensive effects of GLP-1RAs, including natriuresis, vasodilation, anti-inflammatory properties, and sympathetic nervous system suppression.^20^^,^^47^, ^48^, ^49^, ^50^ Furthermore, semaglutide was frequently associated with a reduction in the intensity of antihypertensive therapy among patients with hypertension, particularly those with resistant hypertension,^16^^,^^17^ which may substantially decrease medication burden and improve treatment adherence.

Experimental studies have long investigated and hypothesized the role of adipocytes in aldosterone synthesis and regulation.^51^, ^52^, ^53^ Patients with obesity-related hypertension or heart failure therefore may derive greater benefit from MRA use due to obesity-induced aldosterone excess. In patients with heart failure and obesity, both those with reduced and preserved ejection fraction, the use of MRAs resulted in a more pronounced risk reduction of cardiovascular outcomes.^54^, ^55^, ^56^ Despite these beneficial effects of MRAs, GLP-1RAs were associated with lower hazards of cardiovascular and renal outcomes and a smaller but significant reduction in BP compared with MRAs among patients with RHTN and overweight or obesity. These findings suggest that GLP-1RA therapy may be considered as a potential alternative or complementary therapeutic option, particularly in case of MRA intolerance or high cardiovascular-kidney-metabolic risk in this patient population.

Subgroup analyses were designed to assess whether treatment associations were consistent across clinically relevant subgroups, not to compare baseline risk across race, age, or BMI categories within a treatment group. PSM was performed within each subgroup to balance baseline characteristics between GLP-1RA and MRA users. Because patients were not matched across subgroup categories, crude event rates across categories should not be interpreted as adjusted comparisons of baseline risk. Apparent differences in subgroup-specific event rates, including findings that may appear counterintuitive, may reflect differences in baseline characteristics, follow-up duration, sample size, event numbers, or other subgroup-specific factors.

The incidence of recorded adverse events was comparable between patients receiving GLP-1RAs and those receiving MRAs, although non-serious gastrointestinal events were numerically more frequent in the GLP-1RA group. Importantly, GLP-1RA use was not associated with a higher risk of serious adverse events, including sepsis or acute pancreatitis.

A key strength of this study is its large sample size derived from real-world clinical data, which enables robust statistical analyses and includes sufficiently diverse subgroups, enhancing the applicability of the results across different populations. In addition, propensity score matching was employed to balance baseline characteristics between GLP-1RA and MRA groups, reducing confounding and allowing the observational design to more closely approximate a randomized controlled trial. Several limitations should still be considered when interpreting these findings. First, the retrospective observational design precludes definitive causal inference. Confounding by indication remains a particular concern because GLP-1RAs and MRAs are prescribed for partly different clinical indications. To mitigate this concern, we restricted the cohort to patients with RHTN and overweight or obesity, excluded patients with heart failure or secondary causes of RHTN, and performed subgroup and sensitivity analyses, which yielded similar results. Residual unmeasured confounding from treatment changes, clinical management, adherence, dosing intensity, or treatment escalation or de-escalation cannot be excluded, although E-value analyses suggested that substantial confounding would be required to fully explain away the observed associations. Differences in treatment access, insurance coverage, affordability, and prescribing practices may also have introduced selection bias. Second, standardized mean differences for several baseline covariates remained above the 0.10 threshold, indicating that residual imbalance may have influenced the observed associations despite adjustment for a broad set of baseline covariates. Nevertheless, an additional Cox regression analysis adjusted for all covariates included in the propensity score model yielded findings consistent with the primary analysis. The full propensity score model output, including individual model coefficients, could not be exported from the TriNetX platform, which limits transparency and reproducibility. Third, limitations inherent to electronic health records-based analyses should be acknowledged. Treatment adherence to GLP-1RAs or MRAs could not be ascertained, and information on dose titration, treatment persistence, and achieved maintenance dose was unavailable. Clinical outcomes were not formally adjudicated and were identified using recorded diagnostic data, which may have introduced outcome misclassification. Missing data could occur due to lack of measurement, incomplete documentation, site-level data availability, or true absence of a recorded clinical fact and therefore cannot be assumed to be missing at random. Center–or organization-level identifiers were unavailable because of data-protection requirements. Consequently, between-center differences and center-level clustering could not be assessed, and standard errors may have been affected if treatment patterns, outcome ascertainment, or patient characteristics varied across centers. Nevertheless, exposure and outcome definitions were applied consistently across both treatment groups. Additionally, the study cohort should not be considered representative of all patients with RHTN. Rather, it represents a selected real-world population of patients with apparent RHTN, overweight or obesity who were eligible for initiation of either GLP-1RA or MRA. Because BMI, BP, and eGFR were required for cohort definition, patients with missing values for these variables were excluded. As missingness cannot be assumed to be missing at random, this may have further affected cohort representativeness and limited generalizability. Nevertheless, the large multicenter real-world data source supports applicability to the defined target population. Furthermore, combining tirzepatide and semaglutide into a single intervention group may have allowed one agent to disproportionately influence the overall estimates. However, a separate analysis comparing the two agents showed similar risk reductions. The exploratory concurrent initiation analysis was limited by smaller sample size, shorter follow-up, and fewer events, precluding definitive conclusions regarding potential additive effects. This study was restricted to patients recorded as biologically female or male because information on gender identity was not available. Finally, the analysis was restricted to the US population, which may limit the generalizability of the findings to other geographic regions or healthcare systems. However, this restriction could not be avoided given the variation in guideline-defined BP thresholds for RHTN across countries.^22^^,^^23^^,^^57^

In conclusion, among patients with resistant hypertension and overweight or obesity, GLP-1RA use was associated with lower risks of cardiovascular and kidney outcomes compared with MRA use in this observational study. GLP-1RAs were also associated with significant BP reduction and exhibited a safety profile comparable to MRAs. These results suggest that GLP-1RAs may be considered as a potential alternative or complementary therapeutic option in this population. Further investigation through randomized controlled trials is needed to confirm these findings and explore the broader clinical implications of GLP-1RA-based therapies in management of hypertension.

## Contributors

ZT participated in research concept, research design, data acquisition, statistical analysis, and drafting of the manuscript. JMW participated in research concept and reviewing contents. KMSO participated in reviewing the contents. AM and BMWS participated in research concept, research design, statistical analysis, supervision, and reviewing the contents. ZT, AM, and BMWS accessed and verified the underlying data.

## Data sharing statement

The data supporting this study's findings are available from TriNetX, LLC, but restrictions apply to their availability. The data were accessed under license and cannot be publicly shared. Accredited researchers may obtain access through a data use agreement with TriNetX, LLC, which may involve licensing fees.

## Declaration of interests

ZT and JMW declare no competing interests. KMSO reports receiving research funding, honoraria or consultancy fees and travel support from German Research Foundation, Urological Research Foundation Berlin, ERA PerMed, COVID-19-Forschungsnetzwerk Niedersachsen, Acadeny2GmbH, Alexion, Alentis, Alnylam, Apellis, Astellas, AstraZeneca, Bayer, BioPorto Diagnostics, Boehringer Ingelheim, Chiesi, CSL Behring, FAST BioMedical, GSK, Novartis, Quark Pharmaceuticals, REATA, Roche, Sanofi, Sobi, Stadapharm, StreamedUp, Vifor Pharma, not related to this article. AM reports receiving lecture fees and honoraria from Daiichi Sankyo and Meta X, not related to this article. BMWS reports receiving lecture fees and honoraria from ADVITOS, Amgen, AstraZeneca, Bayer Vital, Berlin Chemie-Menarini, Boehringer Ingelheim, CytoSorbents, Daichii Sankyo, Miltenyi, Novartis, Pocard, Vifor, not related to this article.

## Appendix A Supplementary data

Supplementary Figures and Tables

Translated Abstract
