# Supplementary material for: Comparative effectiveness of GLP-1 receptor agonists versus mineralocorticoid receptor antagonists as fourth-line pharmacological therapy in patients with resistant hypertension and overweight or obesity: a retrospective multicenter cohort study in the USA
Source: eClinicalMedicine. 2026 Aug 29;99:104176. doi: 10.1016/j.eclinm.2026.104176 (PMC13545350; doi:10.1016/j.eclinm.2026.104176)
Supplement: Supplementary file 1 [file mmc1.docx]

**Supplementary Material**

**Comparative Effectiveness of GLP-1 Receptor Agonists versus Mineralocorticoid Receptor Antagonists as Fourth-line Pharmacological Therapy in Patients with Resistant Hypertension and Overweight or Obesity: a retrospective multicenter cohort study in the USA**

| **Supplemental Table 1** | Inclusion and exclusion criteria for glucagon-like peptide-1 receptor agonists | Page 1 |
| --- | --- | --- |
| **Supplemental Table 2** | Inclusion and exclusion criteria for mineralocorticoid receptor antagonists | Page 8 |
| **Supplemental Table 3** | Outcome definitions for primary and secondary outcomes | Page 14 |
| **Supplemental Table 4** | Outcome definitions for adverse events | Page 15 |
| **Supplemental Table 5** | Covariates for propensity score matching | Page 16 |
| **Supplemental Table 6** | Life table for MACE | Page 18 |
| **Supplemental Figure 1** | Changes in Systolic and Diastolic Blood Pressure and Body Mass Index Throughout the Study Period | Page 19 |
| **Supplemental Figure 2** | Forest plot Comparing tirzepatide and semaglutide for clinical outcomes in Resistant Hypertension with Overweight or Obesity | Page 20 |
| **Supplemental Figure 3** | Forest plot Comparing GLP-1RAs plus MRAs and MRAs for clinical outcomes in Resistant Hypertension with Overweight or Obesity | Page 21 |
| **Supplemental Figure 4** | Sensitivity analysis 1-2 | Page 22 |
| **Supplemental Table 7** | Sensitivity analysis 3 | Page 23 |
| **Supplemental Table 8** | Sensitivity analysis 4 | Page 23 |
| **Supplemental Table 9** | Sensitivity analysis 5 | Page 23 |

| **Adult Patients** | |  | **Code Type** | **Code** | **Description** |
| --- | --- | --- | --- | --- | --- |
|  | must have |  | demographics | Age | Age (between 18 and 80 years) |
| **Patients with resistant hypertension** | | | | | |
| **Patients** |  |  | **Code Type** | **Code** | **Description** |
|  | **Resistant hypertension per guideline definition** | | | | |
|  | must have |  | diagnosis | UMLS:ICD10CM:I10 | Essential (primary) hypertension |
|  |  | and any of | medication | NLM:VA:CV800 | ACE INHIBITORS |
|  |  |  | medication | NLM:VA:CV805 | ANGIOTENSIN II INHIBITOR |
|  |  | and | medication | NLM:VA:CV200 | CALCIUM CHANNEL BLOCKERS |
|  |  | and any of | medication | NLM:RXNORM:2409 | chlorthalidone |
|  |  |  | medication | NLM:RXNORM:5487 | hydrochlorothiazide |
|  |  |  | medication | NLM:RXNORM:5764 | indapamide |
|  |  | and any of | laboratory | TNX:9085 | Blood Pressure, Systolic (between 130.00 and 179.00 mm[Hg]) |
|  |  |  | laboratory | TNX:9086 | Blood Pressure, Diastolic (at least 80.00 mm[Hg]) |
|  | date constraint | | The terms in this group occurred between Jun 1, 2017 and Mar 31, 2025 | | |
|  | event relationship | | Any instance of other drugs occurred 6 months before or up to 6 months after the first instance of resistant hypertension | | |
|  | cannot have |  | medication | NLM:RXNORM:49276 | doxazosin |
|  |  | or | medication | NLM:RXNORM:2599 | clonidine |
|  |  | or | medication | NLM:RXNORM:5470 | hydralazine |
|  |  | or | medication | NLM:RXNORM:6984 | minoxidil |
|  |  | or | medication | NLM:ATC:C02AC | Imidazoline receptor agonists |
|  |  | or | medication | NLM:RXNORM:1202 | atenolol |
|  |  | or | medication | NLM:RXNORM:149 | acebutolol |
|  |  | or | medication | NLM:RXNORM:1520 | betaxolol |
|  |  | or | medication | NLM:RXNORM:19484 | bisoprolol |
|  |  | or | medication | NLM:RXNORM:20352 | carvedilol |
|  |  | or | medication | NLM:RXNORM:31555 | nebivolol |
|  |  | or | medication | NLM:RXNORM:6185 | labetalol |
|  |  | or | medication | NLM:RXNORM:6918 | metoprolol |
|  |  | or | medication | NLM:RXNORM:7226 | nadolol |
|  |  | or | medication | NLM:RXNORM:8332 | pindolol |
|  |  | or | medication | NLM:RXNORM:8787 | propranolol |
| **OR** | **Resistant hypertension in medical records** | | | | |
|  | must have |  | diagnosis | UMLS:ICD10CM:I1A.0 | Resistant hypertension |
|  | date constraint | | The terms in this group occurred between Jun 1, 2017 and Mar 31, 2025 | | |
| **Patients prescribed with GLP-1RA at least 8 weeks after resistant hypertension diagnosis and had an eGFR of at least 30 ml/min/1.73 m2** | | | | | |
| **Patients** |  |  | **Code Type** | **Code** | **Description** |
|  | **Resistant hypertension per guideline definition** | | | | |
|  | must have |  | diagnosis | UMLS:ICD10CM:I10 | Essential (primary) hypertension |
|  |  | and any of | medication | NLM:VA:CV800 | ACE INHIBITORS |
|  |  |  | medication | NLM:VA:CV805 | ANGIOTENSIN II INHIBITOR |
|  |  | and | medication | NLM:VA:CV200 | CALCIUM CHANNEL BLOCKERS |
|  |  | and any of | medication | NLM:RXNORM:2409 | chlorthalidone |
|  |  |  | medication | NLM:RXNORM:5487 | hydrochlorothiazide |
|  |  |  | medication | NLM:RXNORM:5764 | indapamide |
|  |  | and any of | laboratory | UMLS:LNC:8480-6 | Blood Pressure Systolic (between 130.00 and 179.00 mm[Hg]) |
|  |  |  | laboratory | UMLS:LNC:8462-4 | Blood Pressure Diastolic (at least 80.00 mm[Hg]) |
|  | date constraint | | The terms in this group occurred between Jun 1, 2017 and Mar 31, 2025 | | |
|  | event relationship | | Any instance of GLP1A occurred at least 1 day after the first instance of resistant hypertension | | |
|  | **GLP-1RA prescription** | | | | |
|  | must have | any of | medication | NLM:RXNORM:2601723 | tirzepatide |
|  |  |  | medication | NLM:RXNORM:1991302 | semaglutide |
|  |  | and any of | laboratory | UMLS:LNC:98979-8 | Glomerular filtration rate/1.73 sq M (CKD-EPI 2021) (at least 30.00 mL/min/{1.73_m2}) |
|  |  |  | laboratory | UMLS:LNC:62238-1 | Glomerular filtration rate/1.73 sq M (CKD-EPI) (at least 30.00 mL/min/{1.73_m2}) |
| **OR** | **Resistant hypertension in medical records** | | | | |
|  | must have |  | diagnosis | UMLS:ICD10CM:I1A.0 | Resistant hypertension |
|  | date constraint | | The terms in this group occurred between Jun 1, 2017 and Mar 31, 2025 | | |
|  | event relationship | | Any instance of Group 4B occurred at least 1 day after the first instance of resistant hypertension | | |
|  | **GLP-1RA prescription** | | | | |
|  | must have | any of | medication | NLM:RXNORM:1991302 | semaglutide |
|  |  |  | medication | NLM:RXNORM:2601723 | tirzepatide |
|  |  | and any of | laboratory | UMLS:LNC:98979-8 | Glomerular filtration rate/1.73 sq M (CKD-EPI 2021) (at least 30.00 mL/min/{1.73_m2}) |
|  |  |  | laboratory | UMLS:LNC:62238-1 | Glomerular filtration rate/1.73 sq M (CKD-EPI) (at least 30.00 mL/min/{1.73_m2}) |
| **Patients not prescribed with GLP-1RA before resistant hypertension diagnosis** | | | | | |
| **Patients** |  |  | **Code Type** | **Code** | **Description** |
|  | **Resistant hypertension per guideline definition** | | | | |
|  | must have |  | diagnosis | UMLS:ICD10CM:I10 | Essential (primary) hypertension |
|  |  | and any of | medication | NLM:VA:CV800 | ACE INHIBITORS |
|  |  |  | medication | NLM:VA:CV805 | ANGIOTENSIN II INHIBITOR |
|  |  | and | medication | NLM:VA:CV200 | CALCIUM CHANNEL BLOCKERS |
|  |  | and any of | medication | NLM:RXNORM:2409 | chlorthalidone |
|  |  |  | medication | NLM:RXNORM:5487 | hydrochlorothiazide |
|  |  |  | medication | NLM:RXNORM:5764 | indapamide |
|  |  | and any of | laboratory | UMLS:LNC:8480-6 | Blood Pressure Systolic (between 130.00 and 179.00 mm[Hg]) |
|  |  |  | laboratory | UMLS:LNC:8462-4 | Blood Pressure Diastolic (at least 80.00 mm[Hg]) |
|  | date constraint | | The terms in this group occurred between Jun 1, 2017 and Mar 31, 2025 | | |
|  | event relationship | | Any instance of GLP1As occurred on or before the first instance of resistant hypertension | | |
|  | **GLP-1RA** | | | | |
|  | cannot have |  | medication | NLM:RXNORM:2601723 | tirzepatide |
|  |  | or | medication | NLM:RXNORM:1991302 | semaglutide |
| **OR** | **Resistant hypertension in medical records** | | | | |
|  | must have |  | diagnosis | UMLS:ICD10CM:I1A.0 | Resistant hypertension |
|  | date constraint | | The terms in this group occurred between Jun 1, 2017 and Mar 31, 2025 | | |
|  | event relationship | | Any instance of Group 6B occurred on or before the first instance of resistant hypertension | | |
|  | **GLP-1RA** | | | | |
|  | cannot have |  | medication | NLM:RXNORM:1991302 | semaglutide |
|  |  | or | medication | NLM:RXNORM:2601723 | tirzepatide |
| **Patients with available blood pressure data at study baseline** | | | | | |
| **Patients** |  |  | **Code Type** | **Code** | **Description** |
|  | must have |  | laboratory | UMLS:LNC:8480-6 | Blood Pressure Systolic (between 130.00 and 179.00 mm[Hg]) |
|  |  | and | laboratory | UMLS:LNC:8462-4 | Blood Pressure Diastolic (at least 80.00 mm[Hg]) |
| **Patients with available BMI data and at least BMI of 27 kg/m2 at study baseline** | | | | | |
| **Patients** |  |  | **Code Type** | **Code** | **Description** |
|  | must have |  | laboratory | TNX:9083 | BMI (at least 27.00 kg/m2) |
|  | date constraint | | The terms in this group occurred between Jun 1, 2017 and Mar 31, 2025 | | |
| **Patients with available Hb1Ac data at study baseline** | | | | | |
| **Patients** |  |  | **Code Type** | **Code** | **Description** |
|  | must have |  | laboratory | TNX:9037 | Hemoglobin A1c/Hemoglobin |
| **Patients with available eGFR data at study baseline** | | | | | |
| **Patients** |  |  | **Code Type** | **Code** | **Description** |
|  | must have | any of | laboratory | UMLS:LNC:98979-8 | Glomerular filtration rate/1.73 sq M(CKD-EPI 2021) |
|  |  |  | laboratory | UMLS:LNC:62238-1 | Glomerular filtration rate/1.73 sq M (CKD-EPI) |
| **Death within 8 weeks after first resistant hypertension diagnosis** | | | | | |
| **Patients** |  |  | **Code Type** | **Code** | **Description** |
|  | **Resistant hypertension per guideline definition** | | | | |
|  | must have |  | diagnosis | UMLS:ICD10CM:I10 | Essential (primary) hypertension |
|  |  | and any of | medication | NLM:VA:CV800 | ACE INHIBITORS |
|  |  |  | medication | NLM:VA:CV805 | ANGIOTENSIN II INHIBITOR |
|  |  | and | medication | NLM:VA:CV200 | CALCIUM CHANNEL BLOCKERS |
|  |  | and any of | medication | NLM:RXNORM:2409 | chlorthalidone |
|  |  |  | medication | NLM:RXNORM:5487 | hydrochlorothiazide |
|  |  |  | medication | NLM:RXNORM:5764 | indapamide |
|  |  | and any of | laboratory | UMLS:LNC:8480-6 | Blood Pressure Systolic (between 130.00 and 179.00 mm[Hg]) |
|  |  |  | laboratory | UMLS:LNC:8462-4 | Blood Pressure Diastolic (at least 80.00 mm[Hg]) |
|  | date constraint | | The terms in this group occurred between Jun 1, 2017 and Mar 31, 2025 | | |
|  | event relationship | | Any instance of Group 11B occurred before or up to 7 days after any instance of resistant hypertension diagnosis | | |
|  | **Mortality records** | | | | |
|  | cannot have |  | demographics | Deceased | Deceased |
|  |  | or | diagnosis | UMLS:ICD10CM:R99 | Ill-defined and unknown cause of mortality |
| **Patients without end stage kidney disease before or at least 10 weeks after first resistant hypertension diagnosis** | | | | | |
| **Patients** |  |  | **Code Type** | **Code** | **Description** |
|  | **Resistant hypertension per guideline definition** | | | | |
|  | must have |  | diagnosis | UMLS:ICD10CM:I10 | Essential (primary) hypertension |
|  |  | and any of | medication | NLM:VA:CV800 | ACE INHIBITORS |
|  |  |  | medication | NLM:VA:CV805 | ANGIOTENSIN II INHIBITOR |
|  |  | and | medication | NLM:VA:CV200 | CALCIUM CHANNEL BLOCKERS |
|  |  | and any of | medication | NLM:RXNORM:2409 | chlorthalidone |
|  |  |  | medication | NLM:RXNORM:5487 | hydrochlorothiazide |
|  |  |  | medication | NLM:RXNORM:5764 | indapamide |
|  |  | and any of | laboratory | TNX:9085 | Blood Pressure, Systolic (between 130.00 and 179.00 mm[Hg]) |
|  |  |  | laboratory | TNX:9086 | Blood Pressure, Diastolic (at least 80.00 mm[Hg]) |
|  | date constraint | | The terms in this group occurred between Jun 1, 2017 and Mar 31, 2025 | | |
|  | event relationship | | The first instance of ESKD occurred before or up to 70 days after any instance of resistent hypertension | | |
|  | **End stage kidney disease** | | | | |
|  | cannot have |  | procedure | UMLS:CPT:1012752 | Hemodialysis Procedures |
|  |  | or | diagnosis | UMLS:ICD10CM:Z99.2 | Dependence on renal dialysis |
|  |  | or | diagnosis | UMLS:ICD10CM:N18.6 | End stage renal disease |
|  |  | or | procedure | UMLS:CPT:1012740 | Dialysis Services and Procedures |
|  |  | or | procedure | UMLS:CPT:1029674 | Dialysis Circuit Procedures |
|  |  | or | procedure | UMLS:ICD9CM:39.95 | Hemodialysis |
| **Patients without pregnancy during the study time** | | | | | |
| **Patients** |  |  | **Code Type** | **Code** | **Description** |
|  | **Resistant hypertension per guideline definition** | | | | |
|  | must have |  | diagnosis | UMLS:ICD10CM:I10 | Essential (primary) hypertension |
|  |  | and any of | medication | NLM:VA:CV800 | ACE INHIBITORS |
|  |  |  | medication | NLM:VA:CV805 | ANGIOTENSIN II INHIBITOR |
|  |  | and | medication | NLM:VA:CV200 | CALCIUM CHANNEL BLOCKERS |
|  |  | and any of | medication | NLM:RXNORM:2409 | chlorthalidone |
|  |  |  | medication | NLM:RXNORM:5487 | hydrochlorothiazide |
|  |  |  | medication | NLM:RXNORM:5764 | indapamide |
|  |  | and any of | laboratory | UMLS:LNC:8480-6 | Blood Pressure Systolic (between 130.00 and 179.00 mm[Hg]) |
|  |  |  | laboratory | UMLS:LNC:8462-4 | Blood Pressure Diastolic (at least 80.00 mm[Hg]) |
|  | date constraint | | The terms in this group occurred between Jun 1, 2017 and Mar 31, 2025 | | |
|  | event relationship | | Any instance of Pregnancy occurred within 6 months before or up to 3 years after any instance of resistant hypertension | | |
|  | **Pregnancy** | | | | |
|  | cannot have |  | diagnosis | UMLS:ICD10CM:O15 | Eclampsia |
|  |  | or | diagnosis | UMLS:ICD10CM:O14 | Pre-eclampsia |
|  |  | or | laboratory | UMLS:LNC:82810-3 | Pregnancy status |
|  |  | or | diagnosis | UMLS:ICD10CM:O00-O9A | Pregnancy, childbirth and the puerperium |
| **Patients without MRA prescription and without other first-line antihypertensive drugs before GLP-1RA** | | | | | |
| **Patients** |  |  | **Code Type** | **Code** | **Description** |
|  | **GLP-1RA prescription without MRA** | | | | |
|  | must have | any of | medication | NLM:RXNORM:2601723 | tirzepatide |
|  |  |  | medication | NLM:RXNORM:1991302 | semaglutide |
|  | cannot have |  | medication | NLM:RXNORM:9997 | spironolactone |
|  |  | or | medication | NLM:RXNORM:298869 | eplerenone |
|  | date constraint | | The terms in this group occurred at any time | | |
|  | event relationship | | The first instance of other drugs occurred within 6 months before or up to 6 months after the first instance of GLP-1RA prescription | | |
|  | **other drugs** | | | | |
|  | cannot have |  | medication | NLM:RXNORM:49276 | doxazosin |
|  |  | or | medication | NLM:RXNORM:2599 | clonidine |
|  |  | or | medication | NLM:RXNORM:5470 | hydralazine |
|  |  | or | medication | NLM:RXNORM:6984 | minoxidil |
|  |  | or | medication | NLM:ATC:C02AC | Imidazoline receptor agonists |
|  |  | or | medication | NLM:RXNORM:1202 | atenolol |
|  |  | or | medication | NLM:RXNORM:149 | acebutolol |
|  |  | or | medication | NLM:RXNORM:1520 | betaxolol |
|  |  | or | medication | NLM:RXNORM:19484 | bisoprolol |
|  |  | or | medication | NLM:RXNORM:20352 | carvedilol |
|  |  | or | medication | NLM:RXNORM:31555 | nebivolol |
|  |  | or | medication | NLM:RXNORM:6185 | labetalol |
|  |  | or | medication | NLM:RXNORM:6918 | metoprolol |
|  |  | or | medication | NLM:RXNORM:7226 | nadolol |
|  |  | or | medication | NLM:RXNORM:8332 | pindolol |
|  |  | or | medication | NLM:RXNORM:8787 | propranolol |
| **Contraindication** | | | | | |
| **Patients** |  |  | **Code Type** | **Code** | **Description** |
|  | cannot have |  | diagnosis | UMLS:ICD10CM:H35.3 | Degeneration of macula and posterior pole |
| **Patients without heart failure** | | | | | |
| **Patients** |  |  | **Code Type** | **Code** | **Description** |
|  | cannot have |  | diagnosis | UMLS:ICD10CM:I50 | Heart failure |
| **Patients without secondary causes of resistant hypertension** | | | | | |
| **Patients** |  |  | **Code Type** | **Code** | **Description** |
|  | cannot have |  | diagnosis | UMLS:ICD10CM:E26 | Hyperaldosteronism |
|  |  |  | diagnosis | UMLS:ICD10CM:C74.10 | Malignant neoplasm of medulla of unspecified adrenal gland |
|  |  |  | diagnosis | UMLS:ICD10CM:D35.00 | Benign neoplasm of unspecified adrenal gland |
|  |  |  | diagnosis | UMLS:ICD10CM:E24 | Cushing's syndrome |
|  |  |  | diagnosis | UMLS:ICD10CM:I70.1 | Atherosklerosis of renal artery |
|  |  |  | diagnosis | UMLS:ICD10CM:G47.30 | Sleep apnea, unspecified |

**Supplemental Table 1 Inclusion and Exclusion Criteria for Glucagon-like Peptide-1 Receptor Agonists**

| **Adult Patients** | |  | **Code Type** | **Code** | **Description** |
| --- | --- | --- | --- | --- | --- |
|  | must have |  | demographics | Age | Age (between 18 and 80 years) |
| **Patients with resistant hypertension** | | | | | |
| **Patients** |  |  | **Code Type** | **Code** | **Description** |
|  | **Resistant hypertension per guideline definition** | | | | |
|  | must have |  | diagnosis | UMLS:ICD10CM:I10 | Essential (primary) hypertension |
|  |  | and any of | medication | NLM:VA:CV800 | ACE INHIBITORS |
|  |  |  | medication | NLM:VA:CV805 | ANGIOTENSIN II INHIBITOR |
|  |  | and | medication | NLM:VA:CV200 | CALCIUM CHANNEL BLOCKERS |
|  |  | and any of | medication | NLM:RXNORM:2409 | chlorthalidone |
|  |  |  | medication | NLM:RXNORM:5487 | hydrochlorothiazide |
|  |  |  | medication | NLM:RXNORM:5764 | indapamide |
|  |  | and any of | laboratory | TNX:9085 | Blood Pressure, Systolic (between 130.00 and 179.00 mm[Hg]) |
|  |  |  | laboratory | TNX:9086 | Blood Pressure, Diastolic (at least 80.00 mm[Hg]) |
|  | date constraint | | The terms in this group occurred between Jun 1, 2017 and Mar 31, 2025 | | |
|  | event relationship | | Any instance of other drugs occurred 6 months before or up to 6 months after the first instance of resistant hypertension | | |
|  | cannot have |  | medication | NLM:RXNORM:49276 | doxazosin |
|  |  | or | medication | NLM:RXNORM:2599 | clonidine |
|  |  | or | medication | NLM:RXNORM:5470 | hydralazine |
|  |  | or | medication | NLM:RXNORM:6984 | minoxidil |
|  |  | or | medication | NLM:ATC:C02AC | Imidazoline receptor agonists |
|  |  | or | medication | NLM:RXNORM:1202 | atenolol |
|  |  | or | medication | NLM:RXNORM:149 | acebutolol |
|  |  | or | medication | NLM:RXNORM:1520 | betaxolol |
|  |  | or | medication | NLM:RXNORM:19484 | bisoprolol |
|  |  | or | medication | NLM:RXNORM:20352 | carvedilol |
|  |  | or | medication | NLM:RXNORM:31555 | nebivolol |
|  |  | or | medication | NLM:RXNORM:6185 | labetalol |
|  |  | or | medication | NLM:RXNORM:6918 | metoprolol |
|  |  | or | medication | NLM:RXNORM:7226 | nadolol |
|  |  | or | medication | NLM:RXNORM:8332 | pindolol |
|  |  | or | medication | NLM:RXNORM:8787 | propranolol |
| **OR** | **Resistant hypertension in medical records** | | | | |
|  | must have |  | diagnosis | UMLS:ICD10CM:I1A.0 | Resistant hypertension |
|  | date constraint | | The terms in this group occurred between Jun 1, 2017 and Mar 31, 2025 | | |
| **Patients prescribed with MRA at least 8 weeks after resistant hypertension diagnosis and had an eGFR of at least 30 ml/min/1.73 m2** | | | | | |
| **Patients** |  |  | **Code Type** | **Code** | **Description** |
|  | **Resistant hypertension per guideline definition** | | | | |
|  | must have |  | diagnosis | UMLS:ICD10CM:I10 | Essential (primary) hypertension |
|  |  | and any of | medication | NLM:VA:CV800 | ACE INHIBITORS |
|  |  |  | medication | NLM:VA:CV805 | ANGIOTENSIN II INHIBITOR |
|  |  | and | medication | NLM:VA:CV200 | CALCIUM CHANNEL BLOCKERS |
|  |  | and any of | medication | NLM:RXNORM:2409 | chlorthalidone |
|  |  |  | medication | NLM:RXNORM:5487 | hydrochlorothiazide |
|  |  |  | medication | NLM:RXNORM:5764 | indapamide |
|  |  | and any of | laboratory | UMLS:LNC:8480-6 | Blood Pressure Systolic (between 130.00 and 179.00 mm[Hg]) |
|  |  |  | laboratory | UMLS:LNC:8462-4 | Blood Pressure Diastolic (at least 80.00 mm[Hg]) |
|  | date constraint | | The terms in this group occurred between Jun 1, 2017 and Mar 31, 2025 | | |
|  | event relationship | | Any instance of MRA occurred at least 1 day after the first instance of resistant hypertension | | |
|  | **MRA prescription** | | | | |
|  | must have | any of | medication | NLM:RXNORM:9997 | spironolactone |
|  |  |  | medication | NLM:RXNORM:298869 | eplerenone |
|  |  | and any of | laboratory | UMLS:LNC:98979-8 | Glomerular filtration rate/1.73 sq M (CKD-EPI 2021) (at least 30.00 mL/min/{1.73_m2}) |
|  |  |  | laboratory | UMLS:LNC:62238-1 | Glomerular filtration rate/1.73 sq M (CKD-EPI) (at least 30.00 mL/min/{1.73_m2}) |
| **OR** | **Resistant hypertension in medical records** | | | | |
|  | must have |  | diagnosis | UMLS:ICD10CM:I1A.0 | Resistant hypertension |
|  | date constraint | | The terms in this group occurred between Jun 1, 2017 and Mar 31, 2025 | | |
|  | event relationship | | Any instance of Group 4B occurred at least 1 day after the first instance of resistant hypertension | | |
|  | **MRA prescription** | | | | |
|  | must have | any of | medication | NLM:RXNORM:9997 | spironolactone |
|  |  |  | medication | NLM:RXNORM:298869 | eplerenone |
|  |  | and any of | laboratory | UMLS:LNC:98979-8 | Glomerular filtration rate/1.73 sq M (CKD-EPI 2021) (at least 30.00 mL/min/{1.73_m2}) |
|  |  |  | laboratory | UMLS:LNC:62238-1 | Glomerular filtration rate/1.73 sq M (CKD-EPI) (at least 30.00 mL/min/{1.73_m2}) |
| **Patients not prescribed with MRA before resistant hypertension diagnosis** | | | | | |
| **Patients** |  |  | **Code Type** | **Code** | **Description** |
|  | **Resistant hypertension per guideline definition** | | | | |
|  | must have |  | diagnosis | UMLS:ICD10CM:I10 | Essential (primary) hypertension |
|  |  | and any of | medication | NLM:VA:CV800 | ACE INHIBITORS |
|  |  |  | medication | NLM:VA:CV805 | ANGIOTENSIN II INHIBITOR |
|  |  | and | medication | NLM:VA:CV200 | CALCIUM CHANNEL BLOCKERS |
|  |  | and any of | medication | NLM:RXNORM:2409 | chlorthalidone |
|  |  |  | medication | NLM:RXNORM:5487 | hydrochlorothiazide |
|  |  |  | medication | NLM:RXNORM:5764 | indapamide |
|  |  | and any of | laboratory | UMLS:LNC:8480-6 | Blood Pressure Systolic (between 130.00 and 179.00 mm[Hg]) |
|  |  |  | laboratory | UMLS:LNC:8462-4 | Blood Pressure Diastolic (at least 80.00 mm[Hg]) |
|  | date constraint | | The terms in this group occurred between Jun 1, 2017 and Mar 31, 2025 | | |
|  | event relationship | | Any instance of MRA occurred on or before the first instance of resistant hypertension | | |
|  | **MRA** | | | | |
|  | cannot have |  | medication | NLM:RXNORM:9997 | spironolactone |
|  |  | or | medication | NLM:RXNORM:298869 | eplerenone |
| **OR** | **Resistant hypertension in medical records** | | | | |
|  | must have |  | diagnosis | UMLS:ICD10CM:I1A.0 | Resistant hypertension |
|  | date constraint | | The terms in this group occurred between Jun 1, 2017 and Mar 31, 2025 | | |
|  | event relationship | | Any instance of Group 6B occurredon or before the first instance of resistant hypertension | | |
|  | **MRA** | | | | |
|  | cannot have |  | medication | NLM:RXNORM:9997 | spironolactone |
|  |  | or | medication | NLM:RXNORM:298869 | eplerenone |
| **Patients with available blood pressure data at study baseline** | | | | | |
| **Patients** |  |  | **Code Type** | **Code** | **Description** |
|  | must have |  | laboratory | UMLS:LNC:8480-6 | Blood Pressure Systolic (between 130.00 and 179.00 mm[Hg]) |
|  |  | and | laboratory | UMLS:LNC:8462-4 | Blood Pressure Diastolic |
| **Patients with available BMI data and at least BMI of 27 kg/m2 at study baseline** | | | | | |
| **Patients** |  |  | **Code Type** | **Code** | **Description** |
|  | must have |  | laboratory | TNX:9083 | BMI (at least 27.00 kg/m2) |
|  | date constraint | | The terms in this group occurred between Jun 1, 2017 and Mar 31, 2025 | | |
| **Patients with available Hb1Ac data at study baseline** | | | | | |
| **Patients** |  |  | **Code Type** | **Code** | **Description** |
|  | must have |  | laboratory | TNX:9037 | Hemoglobin A1c/Hemoglobin |
| **Patients with available eGFR data at study baseline** | | | | | |
| **Patients** |  |  | **Code Type** | **Code** | **Description** |
|  | must have | any of | laboratory | UMLS:LNC:98979-8 | Glomerular filtration rate/1.73 sq M(CKD-EPI 2021) |
|  |  |  | laboratory | UMLS:LNC:62238-1 | Glomerular filtration rate/1.73 sq M (CKD-EPI) |
| **Death within 8 weeks after first resistant hypertension diagnosis** | | | | | |
| **Patients** |  |  | **Code Type** | **Code** | **Description** |
|  | **Resistant hypertension per guideline definition** | | | | |
|  | must have |  | diagnosis | UMLS:ICD10CM:I10 | Essential (primary) hypertension |
|  |  | and any of | medication | NLM:VA:CV800 | ACE INHIBITORS |
|  |  |  | medication | NLM:VA:CV805 | ANGIOTENSIN II INHIBITOR |
|  |  | and | medication | NLM:VA:CV200 | CALCIUM CHANNEL BLOCKERS |
|  |  | and any of | medication | NLM:RXNORM:2409 | chlorthalidone |
|  |  |  | medication | NLM:RXNORM:5487 | hydrochlorothiazide |
|  |  |  | medication | NLM:RXNORM:5764 | indapamide |
|  |  | and any of | laboratory | UMLS:LNC:8480-6 | Blood Pressure Systolic (between 130.00 and 179.00 mm[Hg]) |
|  |  |  | laboratory | UMLS:LNC:8462-4 | Blood Pressure Diastolic (at least 80.00 mm[Hg]) |
|  | date constraint | | The terms in this group occurred between Jun 1, 2017 and Mar 31, 2025 | | |
|  | event relationship | | Any instance of Group 11B occurred before or up to 7 days after any instance of resistant hypertension diagnosis | | |
|  | **Mortality records** | | | | |
|  | cannot have |  | demographics | Deceased | Deceased |
|  |  | or | diagnosis | UMLS:ICD10CM:R99 | Ill-defined and unknown cause of mortality |
| **Patients without end stage kidney disease before or at least 10 weeks after first resistant hypertension diagnosis** | | | | | |
| **Patients** |  |  | **Code Type** | **Code** | **Description** |
|  | **Resistant hypertension per guideline definition** | | | | |
|  | must have |  | diagnosis | UMLS:ICD10CM:I10 | Essential (primary) hypertension |
|  |  | and any of | medication | NLM:VA:CV800 | ACE INHIBITORS |
|  |  |  | medication | NLM:VA:CV805 | ANGIOTENSIN II INHIBITOR |
|  |  | and | medication | NLM:VA:CV200 | CALCIUM CHANNEL BLOCKERS |
|  |  | and any of | medication | NLM:RXNORM:2409 | chlorthalidone |
|  |  |  | medication | NLM:RXNORM:5487 | hydrochlorothiazide |
|  |  |  | medication | NLM:RXNORM:5764 | indapamide |
|  |  | and any of | laboratory | TNX:9085 | Blood Pressure, Systolic (between 130.00 and 179.00 mm[Hg]) |
|  |  |  | laboratory | TNX:9086 | Blood Pressure, Diastolic (at least 80.00 mm[Hg]) |
|  | date constraint | | The terms in this group occurred between Jun 1, 2017 and Mar 31, 2025 | | |
|  | event relationship | | The first instance of ESKD occurred before or up to 7 days after any instance of resistent hypertension | | |
|  | **End stage kidney disease** | | | | |
|  | cannot have |  | procedure | UMLS:CPT:1012752 | Hemodialysis Procedures |
|  |  | or | diagnosis | UMLS:ICD10CM:Z99.2 | Dependence on renal dialysis |
|  |  | or | diagnosis | UMLS:ICD10CM:N18.6 | End stage renal disease |
|  |  | or | procedure | UMLS:CPT:1012740 | Dialysis Services and Procedures |
|  |  | or | procedure | UMLS:CPT:1029674 | Dialysis Circuit Procedures |
|  |  | or | procedure | UMLS:ICD9CM:39.95 | Hemodialysis |
| **Patients without pregnancy during the study time** | | | | | |
| **Patients** |  |  | **Code Type** | **Code** | **Description** |
|  | **Resistant hypertension per guideline definition** | | | | |
|  | must have |  | diagnosis | UMLS:ICD10CM:I10 | Essential (primary) hypertension |
|  |  | and any of | medication | NLM:VA:CV800 | ACE INHIBITORS |
|  |  |  | medication | NLM:VA:CV805 | ANGIOTENSIN II INHIBITOR |
|  |  | and | medication | NLM:VA:CV200 | CALCIUM CHANNEL BLOCKERS |
|  |  | and any of | medication | NLM:RXNORM:2409 | chlorthalidone |
|  |  |  | medication | NLM:RXNORM:5487 | hydrochlorothiazide |
|  |  |  | medication | NLM:RXNORM:5764 | indapamide |
|  |  | and any of | laboratory | UMLS:LNC:8480-6 | Blood Pressure Systolic (between 130.00 and 179.00 mm[Hg]) |
|  |  |  | laboratory | UMLS:LNC:8462-4 | Blood Pressure Diastolic (at least 80.00 mm[Hg]) |
|  | date constraint | | The terms in this group occurred between Jun 1, 2017 and Mar 31, 2025 | | |
|  | event relationship | | Any instance of Pregnancy occurred within 6 months before or up to 3 years after any instance of resistant hypertension | | |
|  | **Pregnancy** | | | | |
|  | cannot have |  | diagnosis | UMLS:ICD10CM:O15 | Eclampsia |
|  |  | or | diagnosis | UMLS:ICD10CM:O14 | Pre-eclampsia |
|  |  | or | laboratory | UMLS:LNC:82810-3 | Pregnancy status |
|  |  | or | diagnosis | UMLS:ICD10CM:O00-O9A | Pregnancy, childbirth and the puerperium |
| **Patients without GLP-1RA prescription and without other first-line antihypertensive drugs before MRA** | | | | | |
| **Patients** |  |  | **Code Type** | **Code** | **Description** |
|  | **MRA prescription without GLP-1RA** | | | | |
|  | must have | any of | medication | NLM:RXNORM:9997 | spironolactone |
|  |  |  | medication | NLM:RXNORM:298869 | eplerenone |
|  | cannot have |  | medication | NLM:RXNORM:2601723 | tirzepatide |
|  |  | or | medication | NLM:RXNORM:1991302 | semaglutide |
|  | date constraint | | The terms in this group occurred at any time | | |
|  | event relationship | | The first instance of other drugs occurred within 6 months before or up to 6 months after the first instance of MRA prescription | | |
|  | **other drugs** | | | | |
|  | cannot have |  | medication | NLM:RXNORM:49276 | doxazosin |
|  |  | or | medication | NLM:RXNORM:2599 | clonidine |
|  |  | or | medication | NLM:RXNORM:5470 | hydralazine |
|  |  | or | medication | NLM:RXNORM:6984 | minoxidil |
|  |  | or | medication | NLM:ATC:C02AC | Imidazoline receptor agonists |
|  |  | or | medication | NLM:RXNORM:1202 | atenolol |
|  |  | or | medication | NLM:RXNORM:149 | acebutolol |
|  |  | or | medication | NLM:RXNORM:1520 | betaxolol |
|  |  | or | medication | NLM:RXNORM:19484 | bisoprolol |
|  |  | or | medication | NLM:RXNORM:20352 | carvedilol |
|  |  | or | medication | NLM:RXNORM:31555 | nebivolol |
|  |  | or | medication | NLM:RXNORM:6185 | labetalol |
|  |  | or | medication | NLM:RXNORM:6918 | metoprolol |
|  |  | or | medication | NLM:RXNORM:7226 | nadolol |
|  |  | or | medication | NLM:RXNORM:8332 | pindolol |
|  |  | or | medication | NLM:RXNORM:8787 | propranolol |
| **Contraindication** | | | | | |
| **Patients** |  |  | **Code Type** | **Code** | **Description** |
|  | cannot have |  | diagnosis | UMLS:ICD10CM:H35.3 | Degeneration of macula and posterior pole |
| **Patients without heart failure** | | | | | |
| **Patients** |  |  | **Code Type** | **Code** | **Description** |
|  | cannot have |  | diagnosis | UMLS:ICD10CM:I50 | Heart failure |
| **Patients without secondary causes of resistant hypertension** | | | | | |
| **Patients** |  |  | **Code Type** | **Code** | **Description** |
|  | cannot have |  | diagnosis | UMLS:ICD10CM:E26 | Hyperaldosteronism |
|  |  |  | diagnosis | UMLS:ICD10CM:C74.10 | Malignant neoplasm of medulla of unspecified adrenal gland |
|  |  |  | diagnosis | UMLS:ICD10CM:D35.00 | Benign neoplasm of unspecified adrenal gland |
|  |  |  | diagnosis | UMLS:ICD10CM:E24 | Cushing's syndrome |
|  |  |  | diagnosis | UMLS:ICD10CM:I70.1 | Atherosklerosis of renal artery |
|  |  |  | diagnosis | UMLS:ICD10CM:G47.30 | Sleep apnea, unspecified |

**Supplemental Table 2 Inclusion anEexclusion Criteria for Mineralocorticoid Receptor Antagonists**

| **MACE** | | | |
| --- | --- | --- | --- |
|  | Diagnosis | UMLS:ICD10CM:I21 | Acute myocardial infarction |
|  | Demographics | Deceased | Deceased |
|  | Diagnosis | UMLS:ICD10CM:R99 | Ill-defined and unknown cause of mortality |
|  | Diagnosis | UMLS:ICD10CM:I46 | Cardiac arrest |
|  | Diagnosis | UMLS:ICD10CM:I22 | Subsequent ST elevation (STEMI) and non-ST elevation (NSTEMI) myocardial infarction |
|  | Diagnosis | UMLS:ICD10CM:I20.0 | Unstable angina |
|  | Diagnosis | UMLS:ICD10CM:I63 | Cerebral infarction |
|  | Diagnosis | UMLS:ICD10CM:I61 | Nontraumatic intracerebral hemorrhage |
| **Mortality** | | | |
|  | Demographics | Deceased | Deceased |
|  | Diagnosis | UMLS:ICD10CM:R99 | Ill-defined and unknown cause of mortality |
| **CV Events** | | | |
|  | Diagnosis | UMLS:ICD10CM:I21 | Acute myocardial infarction |
|  | Diagnosis | UMLS:ICD10CM:I46 | Cardiac arrest |
|  | Diagnosis | UMLS:ICD10CM:I22 | Subsequent ST elevation (STEMI) and non-ST elevation (NSTEMI) myocardial infarction |
|  | Diagnosis | UMLS:ICD10CM:I20.0 | Unstable angina |
|  | Diagnosis | UMLS:ICD10CM:I63 | Cerebral infarction |
|  | Diagnosis | UMLS:ICD10CM:I61 | Nontraumatic intracerebral hemorrhage |
| **AKI** | | | |
|  | Diagnosis | UMLS:ICD10CM:N17 | Acute kidney failure |
| **MAKE** | | | |
|  | Demographics | Deceased | Deceased |
|  | Diagnosis | UMLS:ICD10CM:R99 | Ill-defined and unknown cause of mortality |
|  | Procedure | UMLS:CPT:1012752 | Hemodialysis Procedures |
|  | Procedure | UMLS:ICD9CM:39.95 | Hemodialysis |
|  | Procedure | UMLS:CPT:1012740 | Dialysis Services and Procedures |
|  | Diagnosis | UMLS:ICD10CM:Z99.2 | Dependence on renal dialysis |
|  | Diagnosis | UMLS:ICD10CM:N18.6 | End stage renal disease |
|  | Procedure | UMLS:CPT:90945 | Dialysis procedure other than hemodialysis (eg, peritoneal dialysis, hemofiltration, or other continuous renal replacement therapies) |
|  | Diagnosis | UMLS:ICD10CM:N18.5 | Chronic kidney disease, stage 5 |
|  | Laboratory | UMLS:LNC:62238-1 | Glomerular filtration rate/1.73 sq M.predicted by Creatinine-based formula (CKD-EPI) (at most 14.90 mL/min/{1.73_m2}) |
|  | Laboratory | UMLS:LNC:98979-8 | Glomerular filtration rate/1.73 sq M.predicted by Creatinine-based formula (CKD-EPI 2021) (at most 14.90 mL/min/{1.73_m2}) |

**Supplemental Table 3 Outcome Definitions for Primary and Secondary Outcomes**

| **Gatrointestinal Disorders** | | | |
| --- | --- | --- | --- |
|  | Diagnosis | UMLS:ICD10CM:R11.1 | Vomiting |
|  | Diagnosis | UMLS:ICD10CM:R19.7 | Diarrhea, unspecified |
|  | Diagnosis | UMLS:ICD10CM:K59.00 | Constipation, unspecified |
|  | Diagnosis | UMLS:ICD10CM:R10.13 | Epigastric pain |
| **Infection** | | | |
|  | Diagnosis | UMLS:ICD10CM:N39.0 | Urinary tract infection, site not specified |
|  | Diagnosis | UMLS:ICD10CM:A41.9 | Sepsis, unspecified organism |
|  | Diagnosis | UMLS:ICD10CM:J18 | Pneumonia, unspecified organism |
| **Cholecystitis and Pancreatitis** | | | |
|  | Diagnosis | UMLS:ICD10CM:K85 | Acute pancreatitis |
|  | Diagnosis | UMLS:ICD10CM:K81.0 | Acute cholecystitis |
| **Orthostatic Hypertension** | | | |
|  | Diagnosis | UMLS:ICD10CM:I95 | Hypotension |
|  | Diagnosis | UMLS:ICD10CM:R42 | Dizziness and giddiness |

**Supplemental Table 4 Outcome definitions for Adverse Events**

| **Demographics** | |
| --- | --- |
|  | Age at Index |
|  | White |
|  | Female |
|  | Black or African American |
|  | Male |
|  | Asian |
| **Diagnosis** | |
| **ICD-10-CM-Codes** |  |
| E11 | Type 2 diabetes mellitus |
| E10 | Type 1 diabetes mellitus |
| E66 | Overweight and obesity |
| N18 | Chronic kidney disease (CKD) |
| I50 | Heart failure |
| I20-I25 | Ischemic heart diseases |
| I25 | Chronic ischemic heart disease |
| I48 | Atrial fibrillation and flutter |
| I70.2 | Atherosclerosis of native arteries of the extremities |
| I60-I69 | Cerebrovascular diseases |
| M10 | Gout |
| M06 | Other rheumatoid arthritis |
| E78 | Disorders of lipoprotein metabolism and other lipidemias |
| J44 | Other chronic obstructive pulmonary disease |
| F17 | Nicotine dependence |
| K74.0 | Hepatic fibrosis |
| F10 | Alcohol related disorders |
| I10-I1A | Hypertensive diseases |
| M30-M36 | Systemic connective tissue disorders |
| C00-D49 | Neoplasms |
| I16 | Hypertensive crisis |
| **Medication** | |
| **Medication codes** |  |
| HS501 | INSULIN |
| 1488564 | dapagliflozin |
| 1545653 | empagliflozin |
| 1373458 | canagliflozin |
| 9997 | spironolactone |
| 298869 | eplerenone |
| CV100 | BETA BLOCKERS/RELATED |
| C10AA | HMG CoA reductase inhibitors |
| 83367 | atorvastatin |
| 301542 | rosuvastatin |
| 1191 | aspirin |
| **Laboratory** | |
| 9083 | BMI |
| 9085 | Blood Pressure, Systolic |
| 9086 | Blood Pressure, Diastolic |
| 9063 | C reactive protein [Mass/volume] in Serum, Plasma or Blood |
| 9014 | Hemoglobin [Mass/volume] in Blood |
| 9002 | Cholesterol in LDL [Mass/volume] in Serum or Plasma |
| 9001 | Cholesterol in HDL [Mass/volume] in Serum or Plasma |
| 9037 | Hemoglobin A1c/Hemoglobin.total in Blood |
| 98979-8 | Glomerular filtration rate/1.73 sq M.predicted by Creatinine-based formula (CKD-EPI 2021) |
| 62238-1 | Glomerular filtration rate/1.73 sq M.predicted by Creatinine-based formula (CKD-EPI) |
| LG34557-5 | Albumin/Creatinine [Mass ratio] in Urine |

**Supplemental Table 5 Covariates for Propensity Score Matching**

| Group | Time, weeks | Number at Risk | Cumulative Events | Cumulative Censored | Kaplan-Meier Survival, % | Lower 95% CI, % | Upper 95% CI, % |
| --- | --- | --- | --- | --- | --- | --- | --- |
| GLP-1RAs | 0 | 4153 | 0 | 0 | 100 |  |  |
| GLP-1RAs | 16 | 3481 | 57 | 615 | 98.6 | 98.1 | 98.9 |
| GLP-1RAs | 32 | 2838 | 85 | 1230 | 97.7 | 97.1 | 98.1 |
| GLP-1RAs | 48 | 2201 | 107 | 1845 | 96.8 | 96.2 | 97.4 |
| GLP-1RAs | 64 | 1573 | 120 | 2460 | 96.2 | 95.4 | 96.8 |
| GLP-1RAs | 80 | 950 | 127 | 3076 | 95.6 | 94.7 | 96.3 |
| GLP-1RAs | 96 | 330 | 132 | 4691 | 95 | 94 | 95.8 |
| MRAs | 0 | 4153 | 0 | 0 | 100 |  |  |
| MRAs | 16 | 3437 | 117 | 599 | 97 | 96.4 | 97.5 |
| MRAs | 32 | 2799 | 156 | 1198 | 95.8 | 95.1 | 96.4 |
| MRAs | 48 | 2181 | 174 | 1789 | 95.1 | 94.4 | 95.8 |
| MRAs | 64 | 1561 | 196 | 2397 | 94.1 | 93.2 | 94.8 |
| MRAs | 80 | 951 | 205 | 2997 | 93.3 | 92.4 | 94.2 |
| MRAs | 96 | 347 | 210 | 3596 | 92.6 | 91.6 | 93.5 |

**Supplemental Table 6 Life Table for MACE**

*Abbreviations: CI, confidence interval; GLP-1RA, glucagon-like peptide-1 receptor agonist; MACE, major adverse cardiovascular event; MRA, mineralocorticoid receptor antagonist.*

**Supplemental Figure 1 Changes in Systolic and Diastolic Blood Pressure and Body Mass Index Throughout the Study Period**

*Abbreviations: BMI, body mass index; GLP-1RA, glucagon-like peptide-1 receptor agonist; MRA, mineralocorticoid receptor antagonist.*

**Supplemental Figure 2 Forest Plot Comparing Tirzepatide and Semaglutide for Clinical Outcomes in Resistant Hypertension with Overweight or Obesity**

Results are showed with hazard ratios and 95% confidence intervals.

*Abbreviations: AKI, acute kidney injury; CI, confidence interval; HR, hazard ratio; MACE, major adverse cardiovascular event; MAKE, major adverse kidney event.*

**Supplemental Figure 3 Forest Plot Comparing GLP-1RAs plus MRAs and MRAs for clinical outcomes in Resistant Hypertension with Overweight or Obesity**

Results are showed with hazard ratios and 95% confidence intervals.

*Abbreviations: AKI, acute kidney injury; CI, confidence interval; GLP-1RA, glucagon-like peptide-1 receptor agonist; HR, hazard ratio; MACE, major adverse cardiovascular event; MAKE, major adverse kidney event; MRA, mineralocorticoid receptor antagonist.*

**Supplemental Figure 4 Sensitivity Analysis 1-2**

Sensitivity analyses were conducted: first, the study duration was extended to five years to ensure adequate evaluation of long-term effects; second, patients with heart failure were also included. Results are showed with hazard ratios and 95% confidence intervals.

*Abbreviations: AKI, acute kidney injury; CI, confidence interval; GLP-1RA, glucagon-like peptide-1 receptor agonist; HR, hazard ratio; MACE, major adverse cardiovascular event; MAKE, major adverse kidney event; MRA, mineralocorticoid receptor antagonist.*

| **Outcomes** | **GLP-1 RAs vs. MRAs**  **HR (95% CI)** | **Log-rank *P*** |
| --- | --- | --- |
| **MACE** | 0.77 (0.66,0.90) | < 0.001 |
| **Mortality** | 0.44 (0.29,0.66) | < 0.001 |
| **Cardiovascular Events** | 0.84 (0.71,0.98) | 0.038 |

**Supplemental Table 7 Sensitivity Analysis 3**

A sensitivity analysis was performed without excluding patients who subsequently initiated the comparator therapy. In this analysis, treatment groups were defined according to the initial treatment at the index date, and patients were retained in their original treatment groups regardless of subsequent initiation of the comparator drug.

*Abbreviations: CI, confidence interval; GLP-1RA, glucagon-like peptide-1 receptor agonist; HR, hazard ratio; MACE, major adverse cardiovascular event; MRA, mineralocorticoid receptor antagonist.*

| **Outcomes** | **GLP-1 RAs vs. MRAs**  **HR (95% CI)** | **Log-rank *P*** |
| --- | --- | --- |
| **MACE** | 0.73 (0.61,0.89) | 0.001 |
| **Mortality** | 0.35 (0.21,0.58) | < 0.001 |
| **Cardiovascular Events** | 0.84 (0.65,1.02) | 0.09 |

**Supplemental Table 8 Sensitivity Analysis 4**

we performed a complete-case sensitivity analysis by excluding patients with missing values for any baseline covariates included in the propensity score model.

*Abbreviations: CI, confidence interval; GLP-1RA, glucagon-like peptide-1 receptor agonist; HR, hazard ratio; MACE, major adverse cardiovascular event; MRA, mineralocorticoid receptor antagonist.*

| **Outcomes** | **GLP-1 RAs vs. MRAs**  HR (95% CI) |
| --- | --- |
| **MACE** | 0.62 (0.53, 0.71) |
| **Mortality** | 0.35 (0.25, 0.49) |
| **Cardiovascular Events** | 0.68 (0.57, 0.81) |
| **MAKE** | 0.70 (0.50, 0.98) |
| **AKI** | 0.54 (0.47, 0.63) |

**Supplemental Table 9 Sensitivity Analysis 5**

A sensitivity analysis using multivariable Cox proportional hazards analyses was conducted to evaluate whether residual imbalances after propensity score matching affected our findings. The five separate Cox proportional hazards analyses were adjusted for all covariates included in the propensity score model.

*Abbreviations: AKI, acute kidney injury; CI, confidence interval; GLP-1RA, glucagon-like peptide-1 receptor agonist; HR, hazard ratio; MACE, major adverse cardiovascular event; MAKE, major adverse kidney event; MRA, mineralocorticoid receptor antagonist.*
