# Supplementary material for: Comparative effectiveness of GLP-1 receptor agonists versus mineralocorticoid receptor antagonists as fourth-line pharmacological therapy in patients with resistant hypertension and overweight or obesity: a retrospective multicenter cohort study in the USA
Source: eClinicalMedicine. 2026 Aug 29;99:104176. doi: 10.1016/j.eclinm.2026.104176 (PMC13545350; doi:10.1016/j.eclinm.2026.104176)
Supplement: Supplementary file 2 [file mmc2.docx]

*The following translations in German were submitted by the authors and we reproduce them as supplied. They have not been peer reviewed. Our editorial processes have only been applied to the original abstract in English, which should serve as reference for this manuscript.*

**Zusammenfassung**

**Hintergrund**

Mineralokortikoid-Rezeptor-Antagonisten (MRAs) sind die in den Leitlinien empfohlene Therapie für resistente Hypertonie. Resistente Hypertonie tritt besonders häufig bei Personen mit Übergewicht oder Adipositas auf. Glukagon-like Peptid-1-Rezeptor-Agonisten (GLP-1RAs) führen zu einem relevanten Gewichtsverlust und reduzieren kardiovaskuläre und renale Ereignisse, während sie den Blutdruck moderat senken. Ob GLP-1RAs als alternative therapeutische Strategie bei Patienten mit resistenter Hypertonie und Übergewicht oder Adipositas von Vorteil sind, ist unbekannt. Wir verglichen die Wirksamkeit von GLP-1RAs und MRAs als eine Viertlinientherapie in dieser Patientengruppe.

**Methoden**

In dieser retrospektiven, multizentrischen Kohortenstudie, die Daten aus dem TriNetX US Collaborative Network nutzte, wurden erwachsene Frauen und Männer mit Übergewicht oder Adipositas und resistenter Hypertonie (unkontrollierter Blutdruck trotz einer 3-fachen Therapie mit ACE-Hemmern/AT1-blockern, Kalziumantagonisten und Diuretika) identifiziert, die zwischen dem 01. Juni 2017 und dem 31. März 2025 eine Viertlinientherapie begonnen hatten. Patienten, die orale oder subkutane GLP-1RAs (Semaglutid oder Tirzepatid) oder orale MRAs (Spironolacton oder Eplerenon) initiierten, wurden verglichen. Der primäre Endpunkt war das Auftreten von schwerwiegenden kardiovaskulären Ereignissen (MACE) während einer 2-jährigen Beobachtungszeit. Sekundäre Outcomes umfassten Gesamtmortalität, kardiovaskuläre Ereignisse, renale Outcomes und Blutdruckveränderungen. Die Propensity Score Matching (PSM) wurde verwendet, um die Charakteristika beim Studienbeginn auszugleichen. Die Outcomes wurden mittels Kaplan-Meier-Schätzungen und Cox-Regressionsmodellen analysiert.

**Ergebnisse**

Unter 213.309 geeigneten Patienten begannen 22.694 mit GLP-1RAs und 5.673 mit MRAs. Nach PSM blieben 4.153 Patienten in jeder Gruppe. Während einer medianen Nachbeobachtungszeit von 1,4 Jahren war die Therapie mit GLP-1RAs mit einem geringeren Risiko für MACE (HR 0,63, 95% CI 0,52-0,78), Gesamtmortalität (HR 0,34, 95% CI 0,21-0,55), kardiovaskuläre Ereignisse (HR 0,74, 95% CI 0,59-0,92), schwerwiegende renale Ereignisse (HR 0,64, 95% CI 0,46-0,88) und akute Nierenschädigung (HR 0,62, 95% CI 0,46-0,83) im Vergleich zu MRAs assoziiert. Die systolischen Blutdrucksenkungen nach 12 Wochen waren ähnlich (-5,7 [95% CI -4,0 bis -7,4] mmHg vs. -6,3 [95% CI -4,7 bis -8,0] mmHg).

**Interpretation**

In unserer retrospektiven Studie war die Therapie mit GLP-1RAs bei übergewichtigen oder adipösen Erwachsenen mit resistenter Hypertonie mit einem geringeren kardiovaskulären und renalen Risiko im Vergleich zu MRAs assoziiert, trotz geringerer Blutdrucksenkungen. GLP-1RAs könnten eine potenzielle alternative oder ergänzende therapeutische Option in dieser Patientengruppe darstellen. Prospektive Studien sind notwendig, um zu bestimmen, ob GLP-1RAs in die Behandlungsstrategien für resistenten Bluthochdruck bei adipösen Patienten integriert werden sollten.

**Finanzierung**

Keine
